# Ribonucleic acid as affected by hepatocarcinogenesis.

**DOI:** 10.1038/bjc.1969.74

**Published:** 1969-09

**Authors:** G. N. Dessev, B. M. Mullock, E. Reid, M. K. Turner


					
597

RIBONUCLEIC ACID AS AFFECTED BY HEPATOCARCINOGENESIS

G. N. DESSEV*, BARBARA M. MULLOCK, E. REID AND M. K. TURNERt
From the Wolfson .Bioanalytical Centre, University of Surrey, London, S. W.11i

Received for publication February 11, 1969

RNA is of especial interest in the search for biochemical derangements
underlying neoplasia. Its level in the microsomal fraction usually falls with
hepatocarcinogenesis (Reid, 1962, 1964). No conclusions can be drawn concerning
the level of nuclear RNA, since few workers have studied nuclei of good purity
(Reid, 1965). Nor can firm conclusions be drawn concerning the influence of
hepatocarcinogenesis on the rate of RNA synthesis in vivo, mainly because in
past isotopic work (reviewed by Reid, 1965) there has seldom been a suitably
short post-injection period coupled with examination of acid-soluble precursors as
well as of RNA itself.

The present aim was to find whether hepatocarcinogenesis affects the RNA
"pattern ", as studied by different methods, and the actual rate of RNA synthesis
in vivo. Some of the experiments were done with tumours, notably hepatomas
induced by ethionine and maintained by transplantation. Others were done with
" precancerous " liver from rats fed an azo-dye or ethionine. Work was also done
with a non-carcinogenic agent, a-naphthylisothiocyanate. This causes bile duct
proliferation like that encountered with certain azo dyes.

EXPERIMENTAL

Animnls and injections.-The rats used for the short-term experiments were
usually young-adult albino males, kept on a 20 per cent protein diet and fasted
overnight before killing (Nodes and Reid, 1963). The control rats were restricted
to the food intake of the corresponding experimental rats. The biochemical
results with ethionine were apparently uninfluenced by the use of hooded in place
of albino rats, by substituting control diet for ethionine diet on the penultimate
day, or by omitting the final fast. The dietary levels were 0-25 per cent for
DL-ethionine (from Sigma Chemical Co. Inc., "Grade II "), and 0 075 per cent
for a-naphthylisothiocyanate (Eastman Kodak Inc.) and for the azo-dyes
(Koch-Light Ltd.).

The azo-dye tumours were known to differ sharply from normal liver in
biochemical features such as glucose-6-phosphatase (IUB  3. 1.3.9) activity
(Nodes and Reid, 1963). Most of the ethionine hepatomas (usually " series UB ")
had been transplanted for about 10 generations. They still contained about 70
per cent of the glucose-6-phosphatase activity found in control livers, unlike the
azo-dye hepatomas, but were hardly of "minimal deviation " type. Care was
taken to free excised tumour nodules from any grossly necrotic material.

* Present address: Biochemical Research Laboratory, Academy of Sciences, Sofia, Bulgaria.
t Present address: Biochemistry Unit, Glaxo Research Ltd., Greenford, Middlesex.

I The work was started at the Chester Beatty Research Institute (Institute of Cancer Research:
Royal Cancer Hospital).

598  G. N. DESSEV, BARBARA M. MULLOCK, E. REID AND M. K. TURNER

The Radiochemical Centre (Amersham, England) furnished the radioisotopes,
viz. [6-14C]orotic acid (monohydrate, 64 or 187 ,uCi/mg.-termed [4-14C]orotic
acid in Chemical Abstracts nomenclature) and [32P]orthophosphate (pH 7 solution;
2-10 mCi/mg.). Injections were given intraperitoneally. The doses per 100 g.
body weight were typically 2 ,uCi for orotate or, in the study of phenol-isolated
RNA, 10 /tCi for orotate and 1 mCi for phosphate. The doses were tripled for
rats with hepatomas.

Subcellular fractions by " classical " procedures.-All g values refer to the bottom
of the tube. The tissue sample was homogenized in 5 parts of 0-25 M sucrose
medium and centrifuged for 10 minutes at 600 x g. The pellet was re-homogenized
and re-centrifuged to give the crude nuclear fraction. The cytoplasmic material
was centrifuged for 15 minutes at 10,000 x g (" Angle 13 " centrifuge, M.S.E.
Ltd.) to give a mitochondrial fraction, which was washed once. Most of the lyso-
somes were present in this fraction. The mitochondrial supernatant was centri-
fuged for 90 minutes at 20,000 x g to give a microsomal fraction and a final
supernatant fraction which was almost free of membranous elements. Aliquots of
each fraction and of the homogenate were freed from acid-soluble material by use
of cold 5 per cent (w/v) perchloric acid, and from lipids by use of ethanol: ether:
chloroform (2: 2 : 1, by volume). The residues were dried and analysed, with
counting at infinite thickness. The yields were similar to those reported by Reid
(1964).

Purified nuclei.-With strict adherence to the procedure of Chauveau, Moule
and Rouiller (1956), normal liver furnished nuclei of good preservation and purity.
The criteria routinely employed, as in the experiments of Reid, El-Aaser, Turner
and Siebert (1964), were: (i) the microscopic appearance without staining (phase
contrast) or after toluidine blue staining; (ii) the levels of acid phosphatase and
5'-nucleotidase, these being typically under 2 per cent and 3 per cent respectively
of the total activity of the homogenate per unit of DNA; and (iii) the RNA/DNA
ratio. This ratio was normally 0-17-0-19, which agrees well with the values
reported for nuclei purified in aqueous or non-aqueous media (inter-alia: Reid,
El-Aaser, Turner and Siebert, 1964; Bucher and Swaffield, 1965). Blobel and
Potter (1966) found lower values with a modified Chauveau procedure which was
reckoned to strip off adhering cytomembranes very efficiently. However,
electron microscopy on nuclei as now prepared has shown that cytomembrane
adhesions are virtually absent (M.S.C. Birbeck, unpublished observations).

The RNA/DNA ratio was very consistent in control rats (see heading to
Table I) if care was taken to use the following standard conditions. The initial
homogenization was performed in 20 parts of 2i2 M sucrose, supplemented by
CaCl2 to 5 x 10-5 M. Centrifugation was at 55,000 x g for 50 minutes in an
angle rotor. The supernatant was decanted and discarded. Then, as an important
step, the tube walls were wiped with paper tissue (which removed adhering cell
debris and any nuclei which had not been pelleted), and the walls together with the
surface of the pellet were washed with 0-25 M sucrose.

With hepatomas it was advantageous, in the interests of purity and yield, to
pass the tissue initially through a mincer (with 1 mm. apertures on the plate),
and to use 10-15 rather than 20 parts of the medium.

The radioactivity of defatted and dried pellets obtained from nuclei, or from
the original homogenate, was determined by scintillation counting of extracts
made by dispersing the pellet and subjecting it to two successive treatments with

RNA AS AFFECTED BY CARCINOGENESIS

5 per cent perchloric acid, each for 15 minutes at 80? C. In agreement with
L0vtrup and Roos (1961) and Hutchison, Downie and Munro (1962), these extrac-
tion conditions were found to be a good compromise, giving almost complete
extraction of the DNA without serious destruction of the deoxyribose released
into the hot acid. For actual estimation the method of Burton (1956) gave good
results, with no interference from RNA. Addition of acetaldehyde was unnecessary
provided that the use of top quality acetic acid was avoided.

For estimation of RNA, the conventional orcinol reaction was modified to give
high sensitivity, by the use of 0*25 per cent orcinol and 0 0035 per cent CuCl2. 2H20
in 5*5 N HCI (final concentrations). The tubes were heated in a boiling water
bath for 30 minutes and read at 665 m/t. There was no evidence of hexose
interference. The extent of the interference from DNA in the estimation was
routinely measured using standards containing DNA and RNA together.
Corrections were made for DNA, which gave a colour yield about 7-5 per cent of
that from RNA.

Results obtained with purified nuclei were corrected to 100 per cent recovery
on the basis of DNA estimations on the nuclei and the original homogenate. The
actual recovery of nuclei was typically 40 per cent, except for hepatomas where it
was typically 10-20 per cent.

Sub-fractions from nuclei.-The fractionation procedure (Rendi, 1960) entailed
suspension of the purified nuclei in 0-25 M sucrose containing 10-2 M MgCl2 (approx.
1-3 volumes or, for hepatomas, 0-8 volumes/g. original tissue) and homogenization
in the presence of Lubrol-W (to 0-29 per cent) and of sodium deoxycholate (to
0-57 per cent) at 0-4? C.  Centrifugation for 10 minutes at 12,000 X g gave a
supernatant which was re-centrifuged for 60 minutes at 145,000 x g in the presence
of 5 x 10-2 M MgCl2 to give a detergent supernatant and a detergent high-speed
sediment representing ribonucleoprotein particles (Rendi, 1960). The 12,000 x g
pellet was homogenized in 0 15 M NaCl solution and, after 30 minutes, was centri-
fuged for 10 minutes at 12,000 x g to give a 0-15 M NaCl extract. The resulting
pellet was exposed for at least one hour to M NaCl, re-centrifuged and re-extracted
with M NaCl, furnishing a M NaCl extract and a M NaCl residue. All fractions were
finally treated with perchloric acid to 5 per cent, preceded in the case of the
detergent supernatant by 1 volume of ethanol: ether (1: 1 by volume) to aid
precipitation.

Phenol-isolated RNA.-The tissue was homogenized in 0-25 M sucrose containing
0-025 M KCI, 0.001 M MgCl2, and 0-05 M tris(hydroxymethyl)aminomethane,
pH 7-6 (measured at 20? C.). The homogenate was usually centrifuged to give
subcellular fractions. The nuclear fraction was removed. The cytoplasmic
material was centrifuged according to a scheme (Samarina, 1964; Dessev, Markov
and Tsanev, 1966a, 1966b) which furnishes not only a mitochondrial fraction
(10,000 X g for 10 minutes; fraction discarded) and a microsomal fraction (78,000
X g for 60 minutes) but also a post-microsomal fraction (" light particles ";
200,000 x g for 150 minutes) and a supernatant fraction containing no RNA
larger than 4-5 S.

RNA was isolated from the crude nuclear fraction by the method of Georgiev
and Mantieva (1960), except that the phenol treatment was at 63? C. for 5 minutes.
Other fractions were treated according to Tsanev, Markov and Dessev (1966), with
0-05 M tris(hydroxymethyl)aminomethane, pH 7-6, as the suspending medium for
pellet fractions and with addition of sodium dodecyl sulphate to 1 per cent.

49

599

600 G. N. DESSEV, BARBARA M. MULLOCK, E. REID AND M. K. TURNER

The treatment with water-saturated phenol (pH 6-0, containing 041 per cent
8-hydroxyquinoline) was for 30 minutes at 0.50 C. The aqueous layer was depro-
teinized three times with phenol and finally treated with 2 volumes of ethanol.
The precipitated RNA was freed from contaminants of nucleotide size by passage
through a G-25 Sephadex column. Agar-gel electrophoresis was performed as
described by Tsanev (1965) and Tsanev et al. (1966) with " lonagar No. 2 "
(Oxoid Ltd.) as medium. The transparent gel films finally obtained were scanned
at 260 m,t in a SP. 800 spectrophotometer (Pye-Unicam Instruments Ltd.) with
a scanning adapter. The same instrument was used for densitometry on the radio-
autograph corresponding to each gel film (Tsanev et at., 1966).

In some experiments with [32P]phosphate, the " base composition " of the RNA
was studied. A portion of the RNA from the G-25 column was freed from low
molecular weight RNA (4-5S) by passage through a G-200 column (Dessev et al.,
1966a, 1966b). The resulting RNA, mainly 18S and 28S RNA if derived from cyto-
plasm, was analysed by the method of Katz and Comb (1963), with final measure-
ment of radioactivity.

Acid-soluble nucleotides, in relation to RNA labelling.-Measurements of pool
specific activity were made on UTP itself or else on other uridine nucleotides
which quickly reach isotopic equilibrium with UTP, viz. UDP, UDPacetylgluco-
samine and UDPglucose. The nucleotides were isolated chromatographically
(Hurlbert and Potter, 1954; Nodes and Reid, 1963).

For the aim of relating RNA labelling to pool labelling, a " snapshot " procedure
was adopted. RNA labelling was compared with pool labelling determined
simultaneously on the same tissue sample (usually from a pair of rats). Experi-
mental rats were compared only with controls killed on the same day, in view of
possible day-to-day variability as mentioned by Hurlbert and Potter (1954). In
each series of experiments at least two time points were studied, early enough to
ensure that the precursor specific activity curve had not passed its maximum.

RESULTS

Nucleic Acid Levels

DNA.-The values for mg. of DNA per g. of tissue as given in Table I show the
expected rise with oc-naphthylisothiocyanate. With ethionine, only the tumours
showed a rise.

Cytoplasmic RNA .-Rats fed c-naphthylisothiocyanate or ethionine have shown
almost normal levels of microsomal RNA (Table I). Analyses on mitochondrial
and supernatant fractions (not tabulated) likewise showed no significant effect of
ethionine.

Nuclear RNA.-Analyses on purified nuclei have shown that nuclear RNA
(expressed relative to DNA; Table I) is elevated in the livers of rats fed ethionine
and, more strikingly, in ethionine-induced hepatomas.

DNA and RNA in nuclear sub-fractions.-When the fractionation procedure
described in the Experimental section was applied to control rats, an average of
88 per cent of the nuclear DNA was recovered in the M NaCl extract and a further
7 per cent in the M NaCl residue. Only traces were found in each of the other
sub-fractions. The corresponding figures for rats fed ethionine for several weeks
were 80 per cent and 15 per cent.

RNA AS AFFECTED BY CARCINOGENESIS

C).*

z ;

t

o t
X -

eC) 00

1    0      - -
t-     0o

co 1-     o Ch ?

-H? *6  ?* ?

C?   .. *    *
,4 oo0 r  CII 1

_0 1        _

-

-H

410

r

0)

.

00-400 -

1 -

rH V -H V

*M  4 00 ;4 F

4 - 4

,-s    W"

4      F-4

m          :Y

P-4    14

01 C

0   1

44

.1- 4 - 4 U   .4        C

0 3j

14             2~~~~~~~~~~~~~~~~

o  4             0
_~~~~~~;       0

601

44

as
--
0

0

C)

0
44

0
C)
44

C)
-+

.4

C1)

1.4

C)

CD d

0

t14

EN

*       C )
C.)

) C)

10  A

rl  . o   .  .

?~ -H ? -H ?V

0   0 0

*   . '. .
0)

14

44

P4

4z
04

o4- 4

C0
0U

14

602 G. N. DESSEV, BARBARA M. MULLOCK, E. REID AND M. K. TURNER

The tendency towards a rise in residual nucleic acid after ethionine was more
marked with RNA than with DNA. In controls the extract and residue respec-
tively contained 46 per cent (standard error ?2-3; 9 observations) and 16 per cent
?2-2; 9) of the nuclear RNA. Ethionine-fed rats gave significantly different
values, viz. 34 per cent (?2-5; 3) and 36 per cent (?6-3; 3). Ethionine did not
significantly affect the distribution of RNA amongst the other sub-fractions,
viz, detergent supernatant, detergent high-speed sediment, and 0-15 M NaCl
extract of the detergent 12,000 x g sediment. In controls these sub-fractions
contained 6 per cent, 22 per cent and 10 per cent respectively of the nuclear RNA.

Values similar to those obtained with ethionine feeding were obtained in two
experiments with oc-naphthylisothiocyanate and in a single experiment with
4-dimethylamino-3'-methylazobenzene. Normal values were obtained with
4-dimethylamino-4'-fluoroazobenzene (two experiments), and in cancerous or
hyperplastic nodules such as are listed in Table I. Thus in two experiments with
transplants of ethionine-induced hepatomas the M NaCl extract contained, on
the average, 49 per cent of the nuclear RNA and the residue contained 17 per
cent.

Pattern of RNA labelling amongst isolated fractions

Cytoplasmic particles and supernatant.-The distribution of RNA labelling
within the cytoplasm was not significantly altered by short-term feeding with
a-naphthylisothiocyanate or carcinogenic agents (Table II). Hepatomas showed a
striking shift of the radioactivity from the particulate fractions to the super-
natant fraction.

Nuclear fraction, compared with homogenate.-Short-term feeding of the various
agents was without effect on the proportion of the homogenate labelling attributable
to nuclei. With hepatomas, however, this proportion was depressed in the case
of the crude nuclear fraction, but was markedly increased in the case of purified
nuclei when corrected to a hypothetical quantitative recovery (Table II and Fig. I).
Individual results as given in Fig. 1 for hepatomas show gross " over-recovery"
of the homogenate activity in the nuclei.

Nuclear sub-fractions.-When purified nuclei from control rats were fractionated,
the proportion of the nuclear activity recovered in the 12,000 X g detergent
supernatant was usually under 5 per cent. At the highest it was 15 per cent, at
40 minutes after isotope injection. The proportion in this supernatant, and in
the sub-fractions obtained therefrom by re-centrifugation at 145,000 X g, was
of the normal order in liver from rats fed the different agents and in tumour nodules.
The labelling of the 0-15 M NaCl extract from the 12,000 x g detergent sediment
was likewise relatively low in controls (under 5 per cent) and in the experimental
tissues.

The main site of the radioactivity of the purified nuclei was the M NaCl extract,
in which 70-74 per cent of the label was present at 30-75 minutes after the injec-
tion. The proportion in this extract tended to be lower in the short-term feeding
experiments, with one exception: the value was 82 per cent in an experiment with
4-dimethylamino-4'-fluoroazobenzene. Cholangiomas and hyperplastic nodules
each gave values below 50 per cent, but hepatoma nodules gave values close to
70 per cent as in the controls. The M NaCl residue from controls typically con-
tained almost 20 per cent of the original labelling. The proportion was higher

RNA AS AFFECTED BY CARCINOGENESIS

TABLE II.-Pattern of RNA Labelling Amongst Subcellular Fractions

The rats were given [14C-]orotic acid 30-150 minutes previously. Within this time range there was
some shift of label from the nuclear to the cytoplasmic fraction but little change in the distribution of
label within the cytoplasm, as is evident from the following percentage values in the controls:

Labelling of crude nuclei [and of purified nucleia] as percentage of homogenate: 85 [84] at 30-40

minutes, 73 [81] at 75 minutes, 49 [53] at 150 minutes.

Labelling of mitochondrial fraction as percentage of cytoplasm: 24 at 30-40 minutes, 22 at 75

minutes, 17 at 150 minutes.

Labelling of microsomal fraction as percentage of cytoplasm: 14 at 30-40 minutes, 25 at 75 minutes,

17 at 150 minutes.

Labelling of supernatant fraction as percentage of cytoplasm: 59 at 30-40 minutes, 52 at 75 minutes,

67 at 150 minutes.

In tabulating data for experimental rats it seemed pointless to distinguish the different times, as is
evident for nuclear fractions in Fig. 1. WVhere agents were fed, the trends were unrelated to the dura-
tion of feeding or to the choice of azo-dye. Hepatomas of different types (cf. Table I) gave similar
results.

Experimental relative to control (as unity)

Proportion of homogenate      Proportion of cytoplasmic

labelling recovered in       labelling recovered in

fraction                     fraction

Crude                  Mito-

nuclear    Purified   chondrial  Microsomal Supernatant
Experimental tissue       fraction    nucleia    fraction    fraction   fraction
Liver from rats fed      .    0-99 (2  * 093 (2)     1-05 (2) - 0.90 (2)    1.00 (2)

a -naphthylisothiocyanate
(for 27-29 days)

Liver from rats fed a    . O*99?0 19 1P07?0*07 0*91 ?0*14      1-25+0*16 0 98?0*06

carcinogenic azo-dye          (8)        (4)         (6)         (6)        (6)
(see Table I)

Liver from rats fed      . 1*03+0*05    1*06?0*06  1*48+0*25 0*96+0*15 0*86?0*10

ethionine (see Table I)      (5)         (5)         (5)         (5)        (5)

Hepatomas                 . 0 74?0-04   1-67+0-22  0-83+0-06   0 53?0 03   1-33+0-08

(6; P <0 01) (7;P<0 05) (6;P<0 05)(6;P<0 001)(6;P<0 01)
a Calculated for 100 per cent recovery of homogenate DNA.

for those experimental tissues which gave a sub-normal proportion in the M NaCl
extract. Since hepatomas gave normal values for this residue, and since even
trivial variations in technique can somewhat alter the distribution between the
M NaCl extract and residue, these experiments did not warrant extension.

Phenol-isolated RNA.-RNA isolated by the phenol procedure was subjected
to electrophoresis, giving patterns such as are shown in Fig. 2. The calibration
measurements of Hadjiolov, Venkov and Tsanev (1966) enable an S value to be
assigned to each RNA peak. The amount of RNA in the 4S-5S region (left-hand side
each diagram) was low with microsomal and " post-microsomal " fractions as of
distinct from whole cytoplasm. Some ran at >28S (right-hand side of each
diagram). The prominent E260 peaks at 18S and 28S (under the arrows) were
highly radioactive with the short post-injection times now employed, whereas
the 4S-5S material was only feebly radioactive, at least when orotate rather than
phosphate was used as a precursor. The different species of RNA showed no
obvious change in relative amount with ethionine feeding, or in hepatomas (Fig. 2).
The amount of 18S RNA was typically two-fifths that of 28S RNA.

The labelling of both cytoplasmic and nuclear RNA tended to rise after
ethionine feeding (Table III). Comparison of cytoplasmic 18S with 28S RNA

603

604  G. N. DESSEV, BARBARA M. MULLOCK, E. REID AND M. K. TURNER

1-5_

LU~~~~~~~~~~

1.         * 0            A
<: _ * v

0-5 _v*

X   .                    ~~~~<

O     0

O~~~~~~~~-

CRUDE        PURIFIED NUCLEI
NUCLEAR        cor r. to 100%/
FRACTION        yield of DNA

FIG[. l.-Nuclear labelling relative to homogenate labelling, in control liver and in hepatomas.

The labelling values refer not to specific radioactivity but to the actual amount.

A different symbol is used for each set of experiments, and the time after injection is denoted
by the shape of the symbol:

Triangles or diamonds denote 30-40 minutes, squares or circles denote 75 minutes, and
half-circles denote 150 minutes.

showed no clear selectivity in the rise. The microsomal fraction tended to show
a greater rise than the- post-microsomal fraction.

The hepatoma values for 18S relative to 28S specific activity, measured in
microsomal and post-microsomal fractions, showed no gross differences from the
host liver (Table IV). Post-microsomal relative to microsomal specific activity
tended to be low in hepatomas compared with host liver (Table IV), but was of the
same order at 2 hours as in the normal liver (2 hours) controls of Table III. It
was notably high at 40 minutes both in hepatomas and in host liver.

32p " base comnposition " after ethionine feeding.- Table V shows that in
ethionine-fed rats injected with labelled phosphate there was no change in the
" base composition " of cytoplasmic RNA (18S + 28S) or of nuclear RNA. In
accordance with the results of Dessev et al. (1966a, b) for normal rats which
likewise received the isotope injection 2 hours beforehand, the labelled RNA
appeared to consist partly of DNA-like RNA, the ratio (C + G)/(A + U) being
lower than the ratio (1-65) calculated for ribosomal RNA from extinction
measurements.

RNA AS AFFECTED BY CARCINOGENESIS         605

606 G. N. DESSEV, BARBARA M. MULLOCK, E. REID AND M. K. TURNER

10 0

=   . ~ ~ ~ ~ .

C             .

cs ~I    -

0~~~~~~

o                4-o
l4~~~~~~~~P 0

II               4-I

10          0~~~~

CC

*0.              0

01~~~~X
100  _   I  I

-? -

o  *.i S 1 o Co . '

..10 s  ,  ce  o
_~ .0).

ii ~~~~~~~~~ -~~6

10  ~II.~II  I 8

CC~~ _0 4 1   0C
*  CC Clo1  b    CL,

-  - - -      d01

m   s   _   os  O~~~~~~~~~~~~~~~~~l

-       -  -   Cl)CC

o~ .

*       *  *  C) t

~ _l

CO   m  Ct  | M 4C4t

*       .   .  C;U
01      CC) I-.? =  Q
C)4-    t; 44  e dC

,- o               0 4,

O O Pq00

-4

.5

9

Ca
4-4

0

*-4
.4

CL)

CZ)
-4

C)

C )

to f
._  CC.

0

~4-  010m

CD.
4-4  *,  o

S.

.C)  22

0

-

C) C) -

,,caOO_

o E       C Ea

XC)

-4,- .? C)

0C)
?4't, (s t

C)

o    cd

o

PCa

-C 0-4

CC0

o>+ E C

C         z

Ca
0~

4-4

k 0, ce

~4-4     C

0

o~ e. o ,t

-4Z o

4-D:>*
CD ;  U
4)=   =
* ' de

P -?
4,. - d

E-:i   ,

00
Cl

* -

*.

Ht
?
EHZ

CC

0   -

m

0 1

q

A,o

RNA AS AFFECTED BY CARCINOGENESIS

TABLE IV.-Labelling in Hepatomnas: 18S RNA Compared with 28S RNA

The hepatomas were transplants, originally induced with ethionine. The precursor was orotate in
all experiments. Measurements of radioactivity and extinction were made on the central portion
of electrophoretically separated peaks such as are shown in Fig.'2. The values represent comparisons,
viz. hepatomas/host liver, not absolute values (see heading to Table III). Note that liver from the
hepatoma-bearing rats was the " control " tissue. This " host liver " differs markedly from the
hepatoma in the amount of precursor taken up; thus it would be pointless to tabulate comparisons
of actual specific activity as given in Table III for 18S RNA and 28S RNA individually.-The general
aim is to compare patterns of labelling.

Experimental relative to control (as unity)

Post-microsomal   Specific activity  Specific activity
Time after        Microsomal      specific activity  ratio for 18S,     ratio for 28S,

orotate         specific ratio,      ratio,       post-microsomal:  post-microsomal:
injection         18S: 28S          18S: 28S          microsomal        microsomal

2 hours       .  1*35/1*05a = 1-3   1-3/2-4a = 0.55   1-0/4.la = 0-25  1.05/1.75a = 0-6

(expt. 6)

2 hours       .   1.05/1-45 = 0.7   2-5/2-15 = 1-2    1-7/2-65 = 0-65   0.7/1-75  = 0 4

(expt. 12)

40 minutes    .    2.95/2-0 = 1.35   3.0/2.0 = 1-5    2.95/8.0 = 0-25    2.1/4-95 = 0.45

(expt. 13)

a Ratio for hepatoma/ratio for host liver.

TABLE V.-Base Composition (32p) of Isolated RNA

The whole-cytoplasmic fraction was a pool from the individual rats of experiment 7 in Table JII.
The crude nuclear fractions were similarly pooled, and were treated with phenol under the usual
conditions; the interface material was then treated with phenol at 630 C., to give " nuclear RNA ".
The cytoplasmic RNA was freed from low molecular weight and RNA by use of Sephadex G-200 and
then analysed as stated in the Experimental section. without electrophoretic separation. The
nuclear RNA was purified by use of Sephadex G-25.

"Base composition ", ,umoles/100 ,moles of

2'(3')-ribonucleotide

,~~~~~~~~~~

C + G
Rats           Type of RNA        CMP       AMP       GMP       UMP      A + U
Control  .    .     . Cytoplasmic   .    23 7      22 6      32 2       21 4      1 27
Ethionine-fed  .    . Cytoplasmic   .    22*9      24*0       32 8      20 3      1 25
Control  .    .    . Nuclear        .    24.7      23 9      31 3       19 9      1-28
Ethionine-fed  .   . Nuclear        .    26 3      22-6      30 2       20 6      1 31

RNA labelling relative to " pool " labelling

Validation of procedures.-Rates of RNA synthesis cannot be compared merely
on the basis of RNA labelling, since experimental and control tissues may differ
in the specific activity of the immediate precursor (which is UTP if labelled orotate
is used). Hepatomas necessarily differ from liver in precursor labelling, partly
because the pool of uridine nucleotides is smaller (Nodes and Reid, 1963). Pre-
cancerous liver may likewise be abnormal. The specific activity of UTP within
1-5 hours after [14C]orotate injection was typically 0-8 times that for normal liver
in ethionine-fed rats as now studied and only 0-5 times the normal value in rats
fed a carcinogenic azo-dye.

Precursor measurements are, then, necessary. They should be made earlier
than the peak of precursor labelling if they are to be used in assessing RNA
synthesis. In accordance with findings by Reid and Stevens (1961), the specific
activity of UDP and UTP has shown a peak at 1-2 hours after intraperitoneal

607

608 G. N. DESSEV, BARBARA M. MULLOCK, E. REID AND M. K. TURNER

injection of [14C]orotate. From altogether 21 experiments in pairs of control
rats, the following mean values have now been obtained for the specific activity
of UTDP or UTP, expressed as mIaCi/4amole for an injection of 1 ,tCi/100 g. body
weight: 10 minutes, <35; 20 minutes, 60; 30 minutes, 65; 40 minutes, 71; 90
minutes, 77; 180 minutes, 37; 240 minutes, 53; 510 minutes, 30; 720 minutes, 20.
The trend was similar in the experimental rats, studied for up to 150 minutes.

RNA does not attain maximum labelling within 2 hours, even in the case of the
crude nuclear fraction the RNA of which becomes labelled rapidly (Hurlbert and
Potter, 1954). Accordingly, a measure of the rate of RNA synthesis may be
obtained by relating whole-tissue RNA labelling per unit time (from zero time)
to uridine nucleotide specific activity at the time of killing. The controls in a
series of 21 experiments gave the following mean values for this relationship:
30 minutes, 0 40; 40 minutes, 0 43; 75 minutes, 0 34; 150 minutes, 0 33. The
units are such that these values would represent ,umoles UTP incorporated per
30 minutes into the RNA of 1 g. of liver if the labelling of precursor and products
each rose linearly during the post-injection period. The difference between the
30 minute mean and the 150 minute mean, viz. 0-07?0-06 (standard error), is
not significant. Any lack of constancy could be due to non-linearity in the time
course of precursor labelling. Degradation of certain nuclear RNA fractions
shortly after synthesis could introduce an error, but the total RNA synthesized
in rat liver during a period of the order of an hour after isotope injection is relatively
stable (Potter, Gebert and Pitot, 1966). Calculation of RNA labelling as the
amount of label per g. of tissue, rather than as RNA specific activity, has
the advantage that any difference between experimental and control rats in the
amount of RNA is of no consequence.

It is not claimed that true values for the rate of RNA synthesis have now been
obtained. The values do serve to compare the rates of RNA labelling in experi-
mental and control animals whilst allowing for any inequalities in the labelling
of the immediate precursor of RNA.

In most experiments the RNA was not purified before measurement of its
radioactivity. A contaminant such as UTP might have been present in the
material taken for radioactivity measurement (Kammen, Klemperer and
Canellakis, 1961; Tsanev et al., 1966). To check this possibility, samples of
purified nuclei from rats given labelled orotate (40 minutes beforehand) were
subjected to mild alkaline hydrolysis so as to liberate 2'(3')-ribonucleotides.
After deproteinization, the digest was treated with 0 5 N perchloric acid (15
minutes at 100?) whereby UTP would be converted into 5'-UMP, leaving 2'(3')-
UMP unchanged. These nucleotides were separated by gradient elution chromato-
graphy on Dowex-I resin after addition of unlabelled carrier material. Of the
radioactivity in nuclei from control rats, 96 per cent was recovered in the
2'(3')-UMP, with only 1-3 per cent in the 5'-UMP. With ethionine-fed rats,
102 per cent was recovered in the 2'(3')-UMP and 1-3 per cent in the 5'-UMP.
Since CMP would not be labelled with short post-injection times as now employed,
these recoveries are reassuring.

Relative rates of RNA synthesis.-The approach was as outlined above.
Values obtained for the amount of radioactivity in whole-liver RNA were related
to values obtained for precursor specific activity in the same tissue material, to
give a measure of rate of RNA synthesis. The effect of ethionine feeding in
apparently accelerating RNA synthesis (Turner and Reid, 1964) was manifest even

RNA AS AFFECTED BY CARCINOGENESIS

with only 1 week of feeding (Table VI). With only a single exposure to ethionine,
given in the diet or by injection, there appeared to be a depression of RNA synthesis.

From Table VI it further appears that RNA synthesis is depressed by
a-naphthylisothiocyanate, is unaffected by carcinogenic azo dyes, and is strikingly
accelerated in hepatomas of different types.

Effect of actinomycin and other agents in ethionine-fed rats.-Three agents were
tried in exploratory experiments: actinomycin D, adenine, and puromycin.
The agents were given in 4 doses during the 2 days before killing, to rats of body
weight about 250 g. As before, the measure of RNA synthesis was the labelling
of RNA relative to precursor specific activity. The latter tended to rise with
each of the agents studied.

In single experiments where the time interval from orotate injection was 150
minutes, the relative labelling in ethionine-fed rats fell by 30 per cent when
adenine was given (total dose 80 mg., subcutaneously) and by 17 per cent when
puromycin was given (25 mg., intraperitoneally). With actinomycin ID in low
dosage (total 30 ,ug., intraperitoneally) such that appetite was fairly well main-
tained, the labelling ratio in ethionine-fed rats decreased in each of three experi-
ments-by 30 per cent (at 75 minutes after orotate), by 60 per cent (at 75 minutes)
and by 39 per cent (at 150 minutes) respectively. In none of these experiments
was there a marked change in the distribution of label amongst subcellular
fractions.

Anomalousfindingsfor nuclear RNA.-For the purpose of Table VI, the amount
of RNA radioactivity relative to precursor specific activity gave the best measure
of RNA synthesis. However, ratios were also calculated with RNA specific activity
as the numerator. An interesting anomaly was thereby found with purified nuclei,
the RNA of which is small in amount and is highly labelled. If one-quarter of the
nucleotide residues in nuclear RNA consist of UMP, and if the unlikely assumption
is made that this UMP equilibrates completely with the UTP precursor soon after
orotate injection, then the upper limit would be 0-25 for the specific activity ratio
expressed as ,tmoles of UTP incorporated per ,ug.-atom of RNA phosphorus. A
more realistic assumption, which would furnish a lower value for the limit, is that
nuclear RNA, in the course of gaining its label from UTP which is initially of lower
specific activity than that observed when the rat is killed, is losing its label by
translocation of RNA to the cytoplasm and possibly by catabolism.

The observed values for this ratio with purified nuclei from controls ranged
from 0-18 at 30 minutes after injection to 0-29 to 150 minutes. The values were
of the same order or slightly lower for rats fed an azo-dye or o-naphthylisothio-
cyanate, or for hepatomas. Ethionine-fed rats gave the remarkably high value
of 0 59 at 150 minutes after injection. The fraction extractable by M NaCl from
the 12,000 X g detergent pellet typically gave a value one-third higher than for
whole nuclei.

DISCUSSION

Liver from rats fed azo-dyes. In the literature on RNA synthesis as affected
by azo-dyes (Reid, 1962, 1965), Kono's work (1964) is the most relevant. Contrary
to his observation of an apparent fall in RNA synthesis, no change has now been
seen. Examination of nuclear RNA, and particularly of the residual RNA
remaining after M NaCl extraction, has shown no consistent effect of azo-dye

609

610 G. N. DESSEV, BARBARA M. MULLOCK, E. REID AND M. K. TURNER

C)~  ci7^

0 o O
>) *

0

4 D

0 C  C _4 )

m ca3E

4 ;.    0

o

4-)

0

44)  '" ~

0)

0)

0.

0)  0

bf0

I.  U

0A

> 4     r

o  4  -

X  >3 Q

0

d

._
.0

012

CC -4

C)C)
;0

00r
r_

0O
0Q

.E@ O  O   *

0H1V0. -H

IS-

O

CC

O c3O

_I

m
00

0t

.O
S

0

4    0   = r- 0
0         M0    010)q

- 0 1   01

as 0      ? all  | X
w: O 4 C C C

0D

0

PCa

0-

N4

0

C)
0

10

0

1i

I_

r0

0')

rt

._

0

I'

EA

m

0

0

N

C)
0

0
0
to

.5

C)

C)

0

Ca

0

C0

I._

0   -$     e

00  CD    CX

CO rt

10  -

rm 10

01 i

-4 -

01
CO

H O    o V
Ii JAiCO C
*  .^   l  <zt

rl- O

O01 1O

I     I

I lto -

-- t  -  01q

c)

0

.a
9

0

r-:

0

tl

0

O

0

N
0

01)

o

S

0

C.Y

w
a)

D

0w 0

0     X
a 05)

LS  &.0

N..O t

4;

0

S

0

0

a

0
04
120
00
120
0 r1

C3 ;r,

~ O

r_) w

w  4'

_ 0

0       -4 rH e  _I -0 C C C r- 4

-_ U  d 0  C O   CO10  m  a Id  I

0 C- O  allI  to  0  o 14r  00 =  l

*: .:  . > . i .~  .~  .~  *   * *

I

RNA AS AFFECTED BY CARCINOGENESIS

feeding. Here there is no disagreement with past work (Kono, 1964; Sporn
and Dingman, 1966; Kizer and Clouse, 1968).

Liver from rats fed ax-naphthylisothiocyanate. This non-carcinogenic agent
raised the DNA concentration, as was expected in view of the report by Lopez
and Mazzanti (1955), now confirmed, that it causes small cells of bile-duct type
to proliferate. Although this proliferation must entail some RNA synthesis, it
does not call into question the present conclusion that, contrary to the view of
Kono (1964), RNA synthesis may be somewhat depressed in the liver as a whole.
RNA synthesis in the liver mainly reflects day-to-day functions performed by
parenchymal cells. These cells may be adversely affected by a-naphthylisothio-
cyanate (Phillips and Steiner, 1964).

Liver from rats fed ethionine.-The lack of a rise in DNA is in accord both with
published biochemical observations (Smith and Salmon, 1964) and with histological
observations. The latter disclosed no bile-duct proliferation, in agreement with
Ito, Marugami, Nakamura, Sasaki, Okajuma and Kitamura (1962). The lack of
a fall in microsomal RNA undermines an earlier conclusion, based mainly on
azo-dye studies (Reid, 1962, 1964), that such depletion may be a consistent
feature of hepatocarcinogenesis.

Ethionine given in a single dose has been reported to depress RNA synthesis,
particularly in the nuclear fraction (Villa-Trevino, Shull and Farber, 1966). This
depression, shown in female rats, has now been confirmed in two experiments with
male rats, but is unlikely to be relevant to carcinogenesis. In contrast, chronic
treatment has been found to accelerate RNA synthesis (Turner and Reid, 1964).
This finding has now been confirmed, subject to a reservation discussed below
concerning the precursor pool. Transcriptase activity shows a rise which may
in part explain this acceleration (Turner and Reid, 1964). From observations
now made in ethionine-fed rats it is inferred that the RNA template operating
in vivo is sensitive to actinomycin D, as in regenerating liver (Tsukada and
Lieberman, 1964). RNA synthesis in ethionine-fed rats is likewise sensitive to
adenine-an effect which Farber's group encountered in normal rats or in rats
given an ethionine injection and which " remains as yet unexplained"
(Villa-Trevino et al., 1966; see also Stewart and Farber, 1968).

The rise in RNA labelling after ethionine feeding tended to be lower for
" post-microsomal " RNA (see below, Hepatomas) than for microsomal RNA.
Otherwise there was little selectivity when comparison was made amongst
fractions obtained by differential centrifugation, or between 18S and 28S cytoplas-
mic RNA. For cytoplasmic RNA without distinction between 18S and 28S there
was no change in the c; 32p base composition

The level of nuclear RNA falls after a single dose of ethionine (Stewart and
Farber, 1968), but rises with ethionine feeding. This rise may well be due to the
faster synthesis. It is unlikely that slower catabolism contributes to the rise,
there being an increase in the " free " activity of acid ribonuclease (A. A. El-Aaser
and E. Reid, unpublished experiments). The nuclear RNA rise was especially
marked in the residue from M NaCl extraction. This accords with a statement
by Farber (1963), now confirmed, that nucleoli are larger and more numerous
after ethionine feeding; the M NaCl residue is likely to consist largely of nucleolar
material (inter alia: Kizer et al., 1965). This residual RNA likewise rises after
thioacetamide, a hepatocarcinogen known to cause nucleolar enlargement (Kizer
et al., 1965). However, in agreement with Kizer and Clouse (1968), it is now

611

612 G. N. DESSEV, BARBARA M. MULLOCK, E. REID AND M. K. TURNER

concluded that a rise in this RNA is not a characteristic and specific feature of
hepatocarcinogenesis.

Hepatomas.-The ratio of nuclear RNA to DNA was much higher in ethionine-
induced hepatomas than in liver from rats fed ethionine for a few weeks. This
rise was not confined to the sub-fraction supposedly of nucleolar origin, although
nucleoli are commonly larger and more numerous in hepatomas induced by ethio-
nine (Farber, 1963) or by other agents. Moreover, the scanty literature on purified
nuclei from hepatomas (Reid, 1965), together with a result now obtained for an
azo-dye hepatoma, indicates that nuclear RNA is not invariably high in hepatomas.

One practical difficulty in isolating nuclei is that yield has to be sacrificed for
the sake of purity. Non-random selection may occur, especially with hepatomas.
This would explain the over-recovery of the hepatoma homogenate labelling in
purified nuclei if corrected to 100 per cent yield on a DNA basis. The inference
that the specific activity of the RNA is higher in the nuclei now isolated than in
the general population of nuclei has support from published work on DNA.
Nuclei isolated by procedures similar to those now used have given particularly
high values for DNA content (Fisher, Holbrook and Irvin, 1963; normal liver)
and DNA specific activity (Niehaus and Barnum, 1964; regenerating liver). As
judged by values for crude nuclear fractions, where the problem of selection does
not arise, the contribution of nuclei to the RNA labelling of homogenates is low
in hepatomas.

There is a need for a closer study of the rapidly labelled RNA of hepatomas,
particularly the RNA which remains unsedimented when the microsomal material
is centrifuged down (Reid and Stevens, 1961). This RNA shows relatively high
labelling in hepatomas. Possibly this RNA may have leaked from the nucleus
during homogenization. With orotate as precursor, the observed labelling could
not have been due to terminal exchange in the soluble RNA, but use of this
precursor precluded examination of the " DNA-like " 18S RNA (detectable from
" 32p base ratios ") that is not readily sedimentable in normal liver (Dessev et al.,
1966a, b). The rapidly labelled post-microsomal RNA of hepatomas may be
in ribonucleoprotein particles that serve as ribosomal precursors.

Normally the specific activity of post-microsomal RNA exceeds that of micro-
somal RNA soon after isotope injection, and the specific activity of 18S RNA
exceeds that of 28S RNA (Dessev et al., 1966a, b). Qualitatively, the labelling
patterns of the hepatoma fractions resemble those for liver, particularly if com-
parison is made with normal rather than host liver. Viewed quantitatively, the
results point to acceleration of the formation of rapidly labelled 18S RNA, perhaps
with a specific activity peak earlier than 40 minutes and with faster transfer
from post-microsomal to microsomal elements. Labelling of 18S RNA seems
to occur faster in regenerating liver (Fausto and van Lancker, 1968), with no
sharp change in the distribution of label amongst the classical sub-cellular
fractions (Fujioka, Koga and Lieberman, 1963). For hepatomas there is little
literature of relevance to the present study of rapidly labelled RNA. As is evident
from Fig. 2, no support has been obtained for the supposed presence in hepatoma
cytoplasm of species of RNA that are normally confined to the nucleus (Drews,
Brawerman and Morris, 1968).

It appears that in hepatomas, as in regenerating liver (Fujioka et al., 1963),
there is accelerated synthesis of RNA in the tissue as a whole. This accords with
certain observations in the scanty literature (Reid, 1958; Munro and Clark, 1959).

RNA AS AFFECTED BY CARCINOGENESIS

RNA catabolism likewise seems to be increased, as judged by the increase in
" free " acid ribonuclease activity observed in the supernatant fraction (Nodes
and Reid, 1963; Roth, Hilton and Morris, 1964). Presumably there is faster
turnover of RNA.

The conclusions about RNA synthesis must be regarded as tentative, in view
of an anomaly now encountered. In hepatoma and liver samples, the specific
activity of RNA from purified nuclei was so high relative to that of its supposed
precursor as to suggest the presence of a cellular compartment containing precursor
material of notably high specific activity. On quantitative grounds this anomaly
could not be due merely to the inadvertent selection of nuclei with exceptionally
high RNA labelling. Uridine nucleotide specific activity in " non-aqueous "
nuclei was sometimes higher than, but nevertheless reflected, the value in whole
tissue, viz. normal liver, liver from rats fed an azo-dye, and hepatomas (Reid,
El-Aaser and Siebert, 1964). High values for the specific activity of RNA relative
to that of total liver UTP have not been observed in experiments where orotate
was injected intravenously rather than intraperitoneally (Fujioka et al., 1963;
Tsukada and Lieberman, 1964; Bucher and Swaffield, 1965). Nevertheless the
possibility that there may be a compartment has been recognized (Bucher and
Swaffield, 1965), and has support from other isotopic work (Waelach, 1962;
Garfinkel and Lajtha, 1963; Kimball and LePage, 1963).

SUMMARY

RNA has been studied in hepatomas and in liver from rats given a dietary
agent-a carcinogenic azo-dye, or ethionine, or a-naphthylisothiocyanate (which
induced bile duct proliferation but is non-carcinogenic).

In ethionine-induced hepatomas and in ethionine-fed rats the ratio of nuclear
RNA to DNA was increased. The rise was most marked in a fraction insoluble
in M NaCl. Neither this change, nor the fall in microsomal RNA as found with
azo-dyes but not with ethionine, can be regarded as important in hepatocarcino-
genesis. When a labelled precursor (orotate) was injected, hepatomas gave a
supernatant fraction with abnormally high RNA labelling relative to that of the
crude-nuclear and other particulate fractions. Purified as distinct from crude
nuclei showed notably high labelling in hepatomas, not confined to any one nuclear
sub-fraction. The isolated nuclei were apparently an unrepresentative selection.

Electrophoresis of RNA from tissue fractions gave 18S and 28S RNA. The
relative specific activity (1 8S: 28S) was within normal limits after ethionine
feeding or in hepatomas. In hepatomas, as in liver, post-microsomal RNA was
more highly labelled than microsomal RNA soon after isotope injection. The
" 32p base composition " was normal in nuclear and cytoplasmic RNA from
ethionine-fed rats.

Comparison of RNA labelling with precursor (uridine nucleotide) labelling
in the whole tissue pointed to changes in the rate of RNA synthesis. The rate
fell with ac-naphthylisothiocyanate. It rose with ethionine if fed for a week or
more, but fell after acute treatment. It rose markedly in hepatomas. Actino-
mycin D depressed the rate in ethionine-fed rats. Nuclear RNA in normal and
experimental rats had so high a specific activity as to suggest that there exists
a special nucleotide compartment readily penetrable by intraperitoneally injected
orotate.

613

614 G. N. DESSEV, BARBARA M. MULLOCK, E. REID AND M. K. TURNER

Grants from the Medical Research Council and from the British Empire
Cancer Campaign for Research supported the work, including initial experiments
done at the Chester Beatty Research Institute (Institute of Cancer Research:
Royal Cancer Hospital). The Institute received a grant from the National
Cancer Institute, U.S. Public Health Service (Research Grant No: CA-03188-09).
The work at the University of Surrey was also helped by the Wellcome Trust and
by Faculty funds. M.K.T. had a Studentship stipend from the Institute of
Cancer Research and later from the University of Surrey. Dr. J. Chauveau
kindly showed M.K.T. his method for preparing nuclei. Mr. C. J. Smith and Mr.
P. Scobie-Trumper gave valuable help with the animals. Actinomycin D was
donated by Merck, Sharpe & Dohme Inc.

REFERENCES

BLOBEL, G. AND POTTER, V. R.-(1966) Science, N. Y., 154, 1662.

BUCHER, N. L. R. AND SWAFFIELD, M. N.-(1965) Biochim. biophys. Acta, 108, 551.
BURTON, K.-(1956) Biochem. J., 62, 315.

CHAUVEAU, J., MOULE, Y. AND ROUILLER, C.-(1956) Expl Cell Res., 11, 317.

DESSEV, G. N., MARKOV, G. G. AND TSANEV, R. G.-(1966a) C.r. Acad. bulg. Sci., 10,

755.-(1966b) Life Sci., 5, 2331.

DREWS, J., BRAWERMAN, G. AND MORRIS, H. P.-(1968) Eur. J. Biochem., 3, 284.
FARBER, E.-(1963) Adv. Cancer Res., 7, 383.

FAUSTO, N. AND VAN LANCKER, J. L.-(1968) Biochim. biophys. Acta, 161, 32.

FISHER, R. F., HOLBROOK, D. J. AND IRVIN, J. L.-(1963), J. Cell Biol., 17, 231.
FUJIOKA, M., KOGA, M., AND LIEBERMAN, I.-(1963) J. biol. Chem., 238, 3401.
GARFINKEL, D. AND LAJTHA, A.-(1963) J. biol. Chem., 238, 2429.

GEORGIEV, G. P. AND MANTIEVA, V. L.-(1960) Biochemistry, N. Y.,26, 103.

HADJIOLOV, A. A., VENKOV, P. V. AND TSANEV, R. G.-(1966) Analyt. Biochem., 17, 263.
HURLBERT, R. B. AND POTTER, V. R.-(1954) J. biol. Chem., 209, 1.

HUTCHISON, W. C., DOWNIE, E. D. AND MUNRO, H. N.-(1962) Biochim. biophys. Acta,

55, 561.

ITO, N., MARUGAMI, M., NAKAMURA, H., SASAKI, S., OKAJUMA, E. AND KITAMURA, H.-

(1962) Gann, 53, 235.

KAMMEN, H. O., KLEMPERER, H. G. AND CANELLAKIS, E. S.-(1961) Biochim. biophys.

Acta, 51, 175.

KATZ, S. AND COMB, D. G.-(1963) J. biol. Chem., 238, 3065.

KIMBALL, A. P. AND LEPAGE, G. A.-(1963) Cancer Res., 23, 1702.
KIZER, D. E. AND CLOUSE, J. A.-(1968) Cancer Res., 28, 502.

KIZER, D. E., SHIRLEY, B. C., Cox, B. AND HOWELL, B. A.-(1965) Cancer Res., 25, 596.
KONO, M.-(1964) Gann, 55, 251.

LOPEZ, M. AND MAZZANTI, L.-(1955) J. Path. Bact., 69, 243.

L0VTRUP, S. AND Roos, K.-(1961) Biochim. biophys. Acta, 53, 1.
MUNRO, H. N. AND CLARK, C. M.-(1959) Br. J. Cancer, 13, 324.

NIEHAUS, W. G. AND BARNUM, C. P.-(1964) J. biol. Chem., 239, 1198.
NODES, J. T. AND REID, E.-(1963) Br. J. Cancer, 17, 745.

PHILLIPS, M. J. AND STEINER, J. W.-(1964) Lab. Invest., 13, 779.

POTTER, V. R., GEBERT, R. A. AND PITOT, H. C.-(1966) Advances in Enzyme Regulation,

4, 247.

REID, E.-(1958) Br. J. Cancer, 12, 428.-(1962) Cancer Res., 22, 398.-(1964) Br. J.

Cancer, 18, 172.-(1965) 'Biochemical Approaches to   Cancer'. Oxford
(Pergamon).

REID, E., EL-AASER, A. A. AND SIEBERT, G.-(1964) Abstracts 6th int. Congr. Biochem.,

p. 328.

RNA AS AFFECTED BY CARCINOGENESIS                     615

REID, E., EL-AASER, A. A., TURNER, M. K. AND SIEBERT, G.-(1964) Hoppe-Seyler's.

Z. physiol. Chem., 339, 135.

REID, E. AND MORRIS, H. P.-(1963) Biochim. biophys. Acta, 68, 647.

REID, E. AND STEVENS, B. M.-(1961) Biochim. biophys. Acta, 49, 215.
RENDI, R.-(1960) Expl. Cell Res., 19, 489.

ROTH, J. S., HILTON, S. AND MORRIS, H. P.-(1964) Cancer Res., 24, 294.
SAMARINA, 0. P.-(1964) Dokl. Acad. Nauk. USSR, 156, 1217.

SMITH, R. C. AND SALMON, W. D.-(1964) Archs Biochem. Biophys., 106, 428.
SPORN, M. D. AND DINGMAN, C. W.-(1966) Cancer Res., 26, 2488.

STEWART, G. A. AND FARBER, E.-(1968) J. biol. Chem., 243, 4479.
TSANEV, R.-(1965) Biochim. Biophys. Acta, 103, 374.

TSANEV, R., MARKOV, G. G. AND DESSEV, G. N.-(1966) Biochem. J., 100, 204.
TSUKADA, E. AND LIEBERMAN, I.-(1964) J. biol. Chem., 239, 2952.
TURNER, M. K. AND REID, E.-(1964) Nature, Lond., 203, 1174.

VILLA-TREViNO, S., SHULL, K. H. AND FARBER, E.-(1966) J. biol. Chem., 241, 4670.

WAELACH, H.-(1962) 'Amino Acid Pools'. Edited by J. T. Holden. Amsterdam

(Elsevier Press), p. 722.

50

				


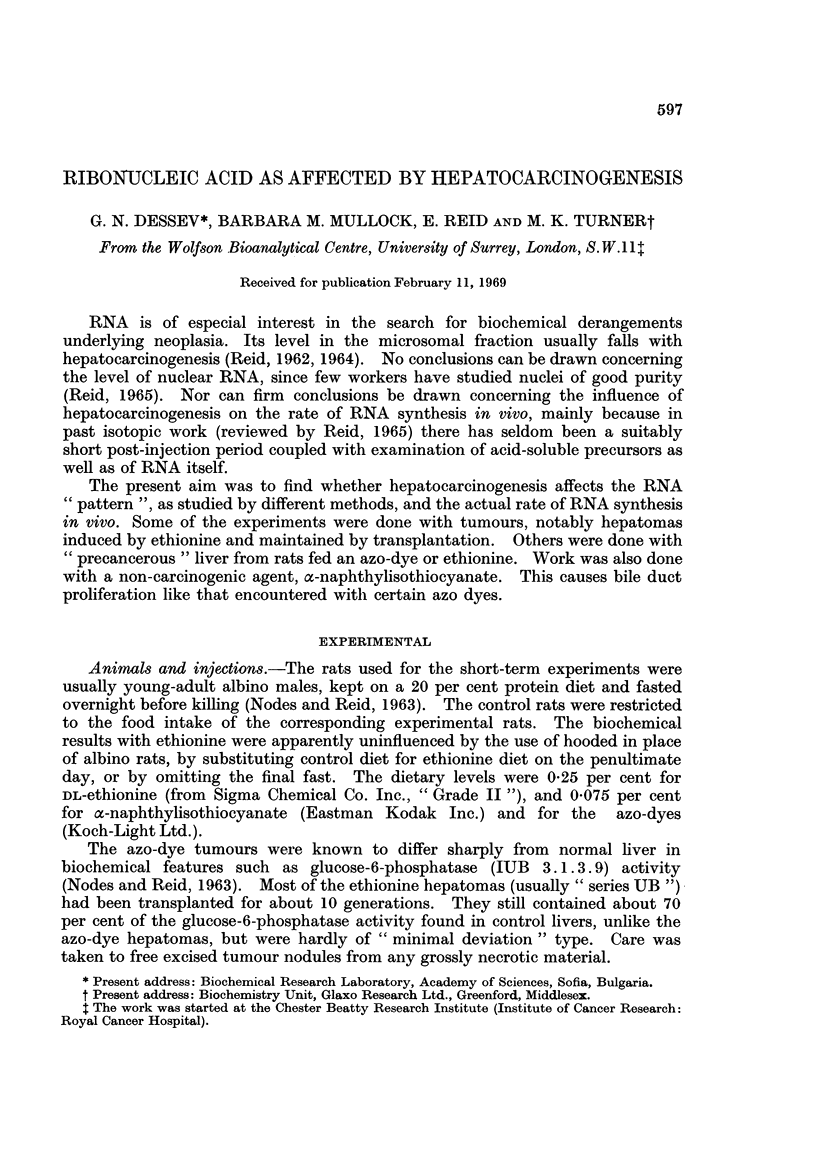

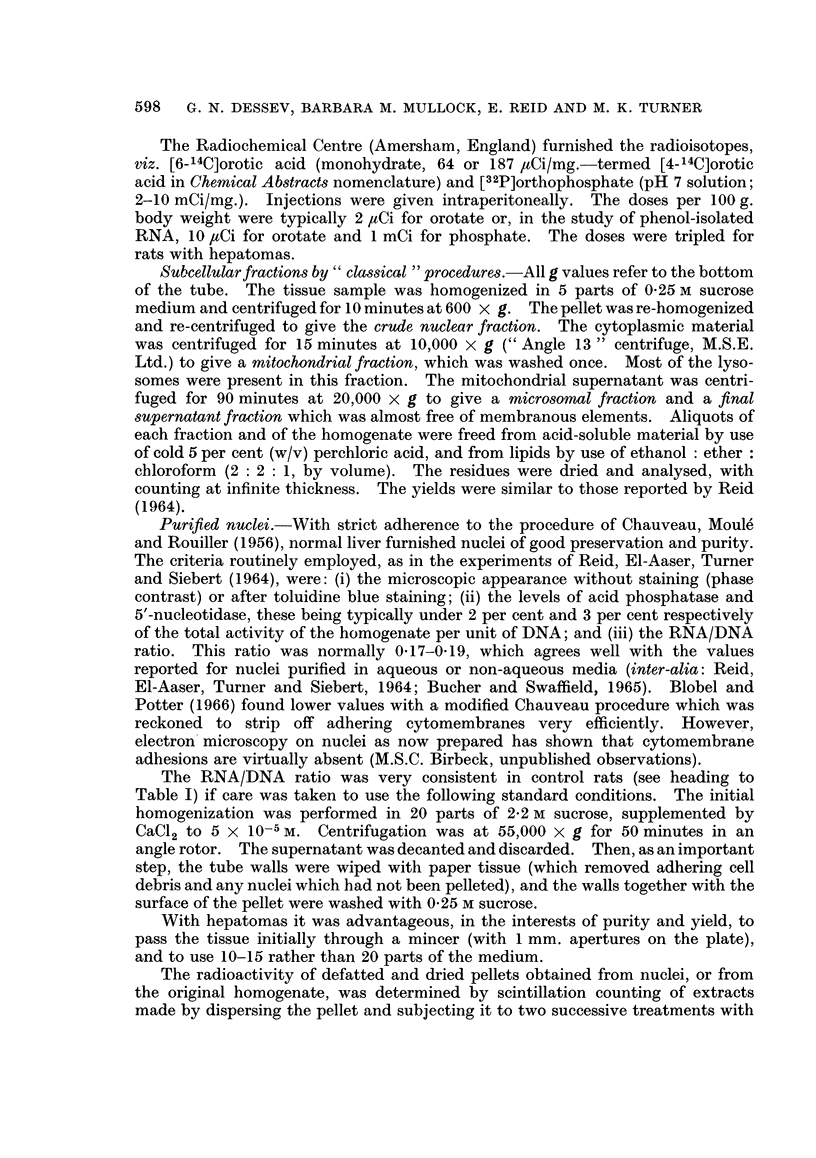

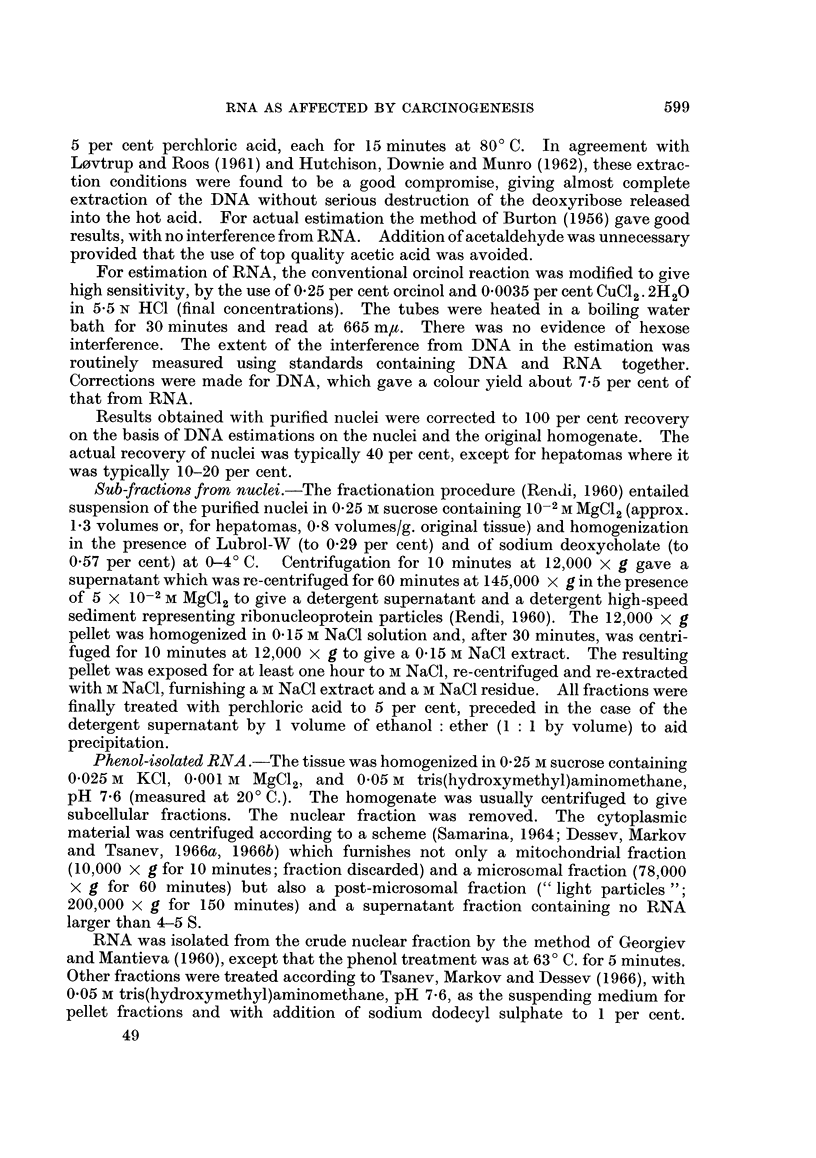

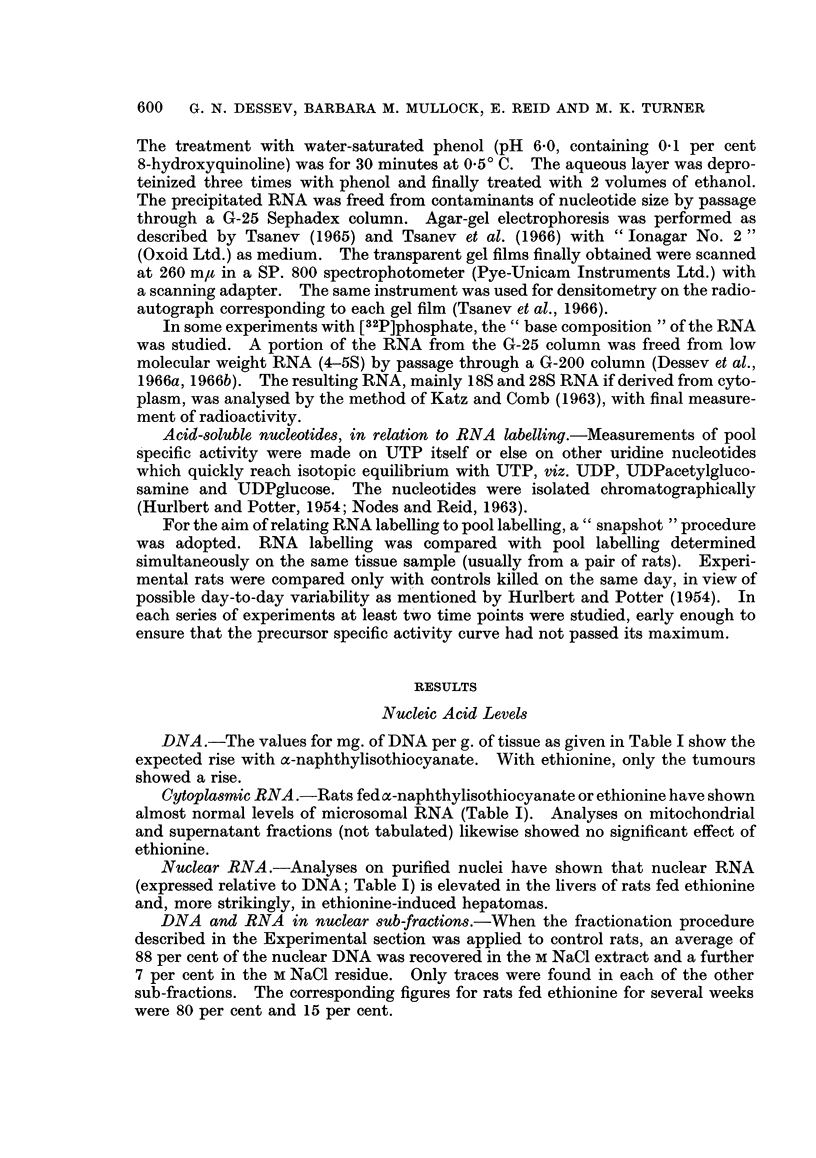

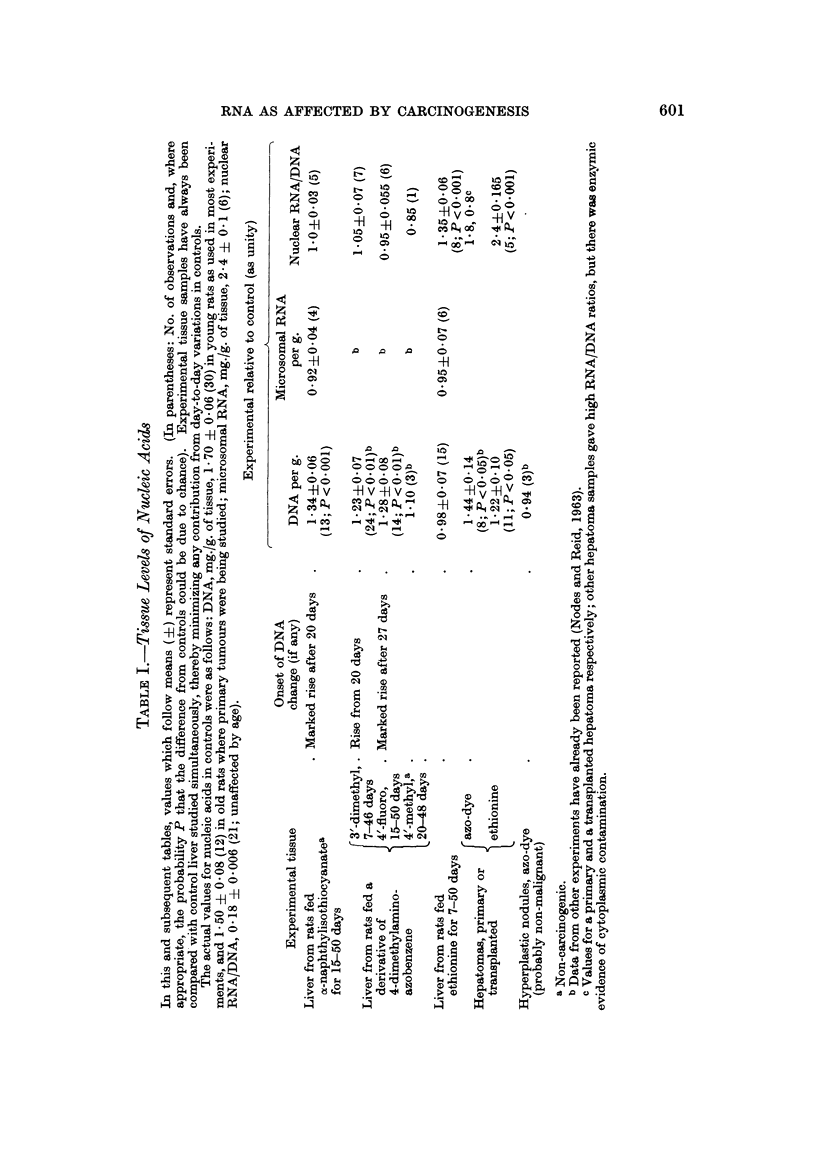

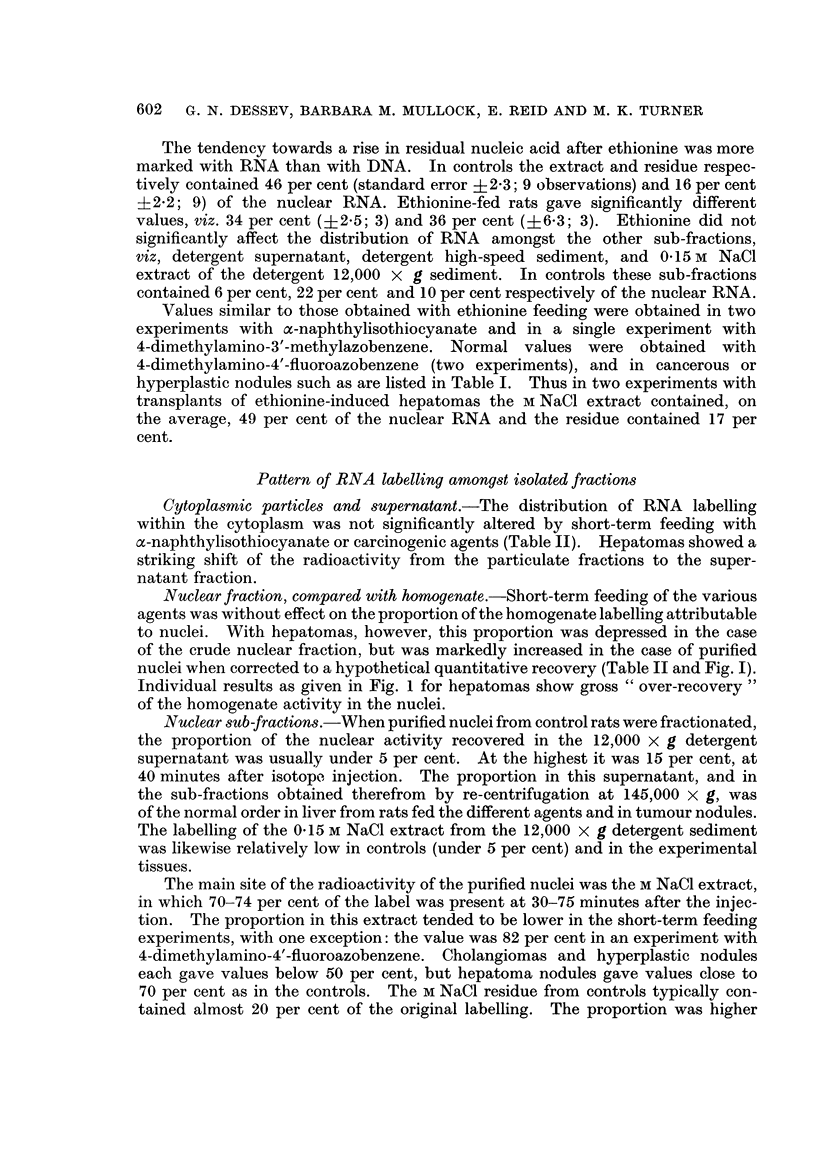

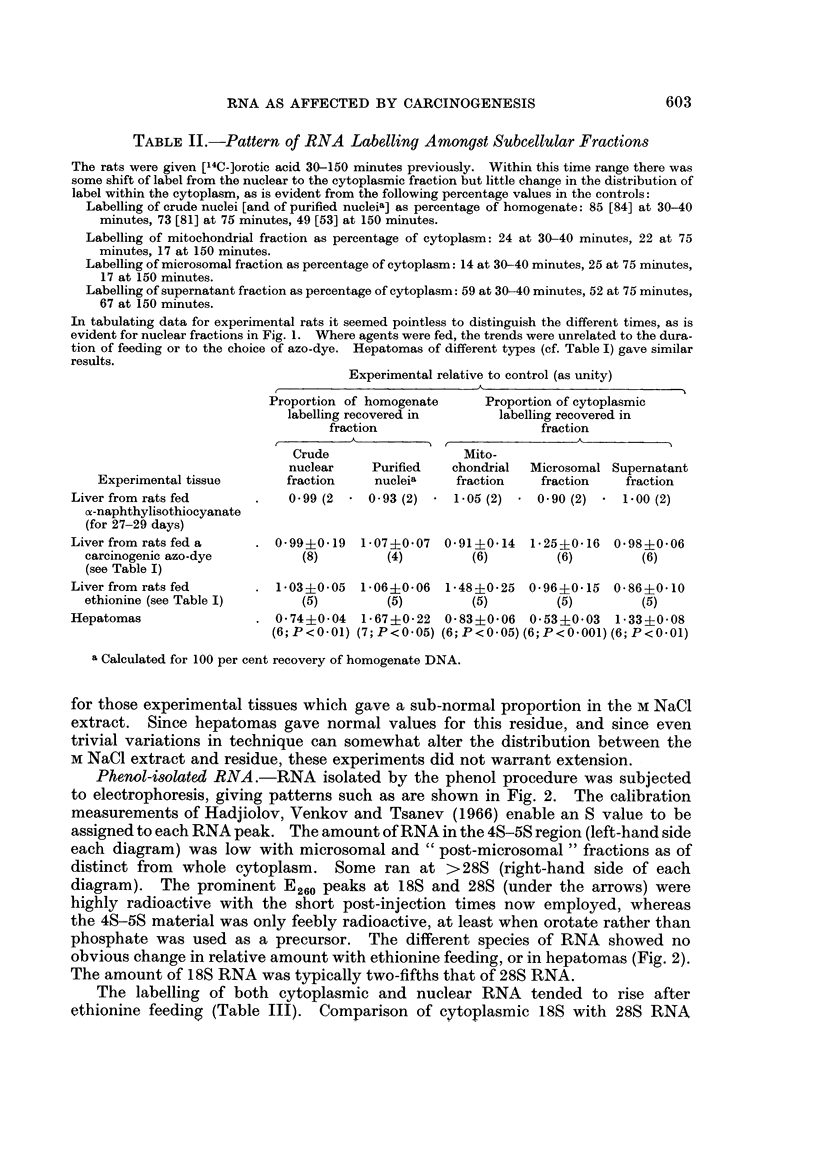

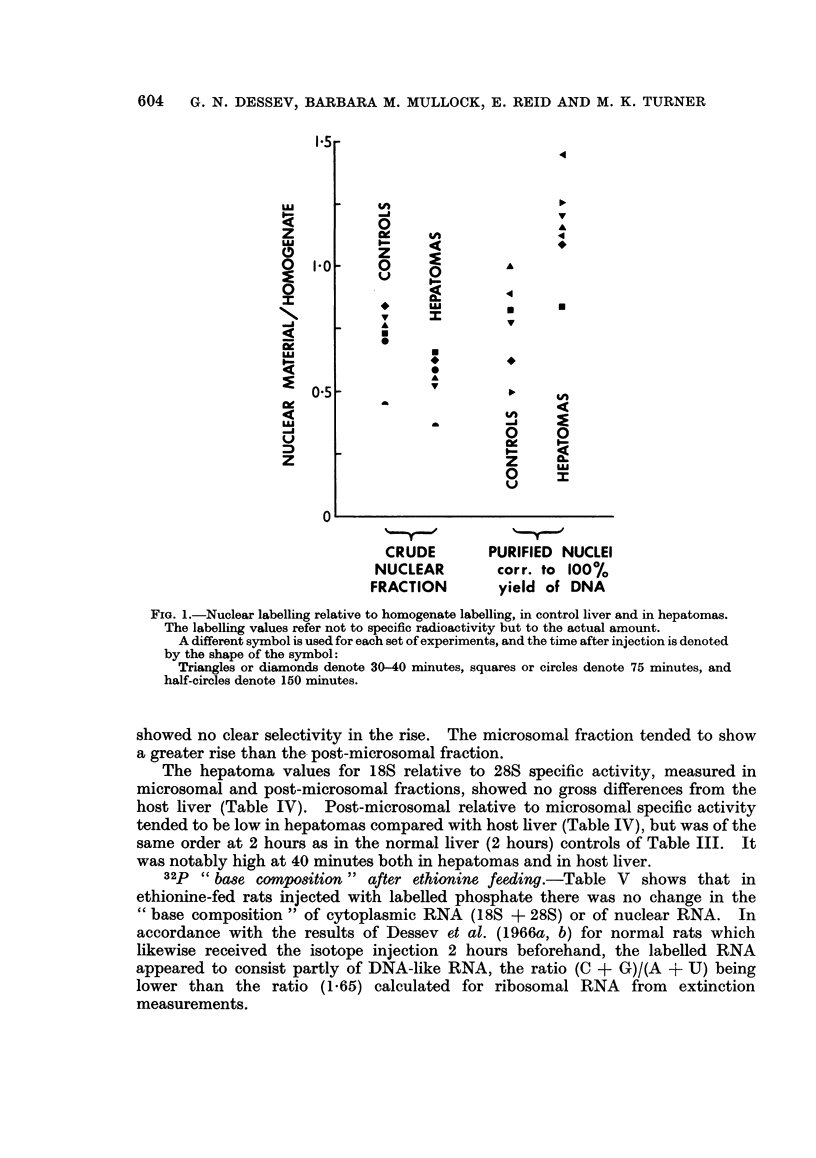

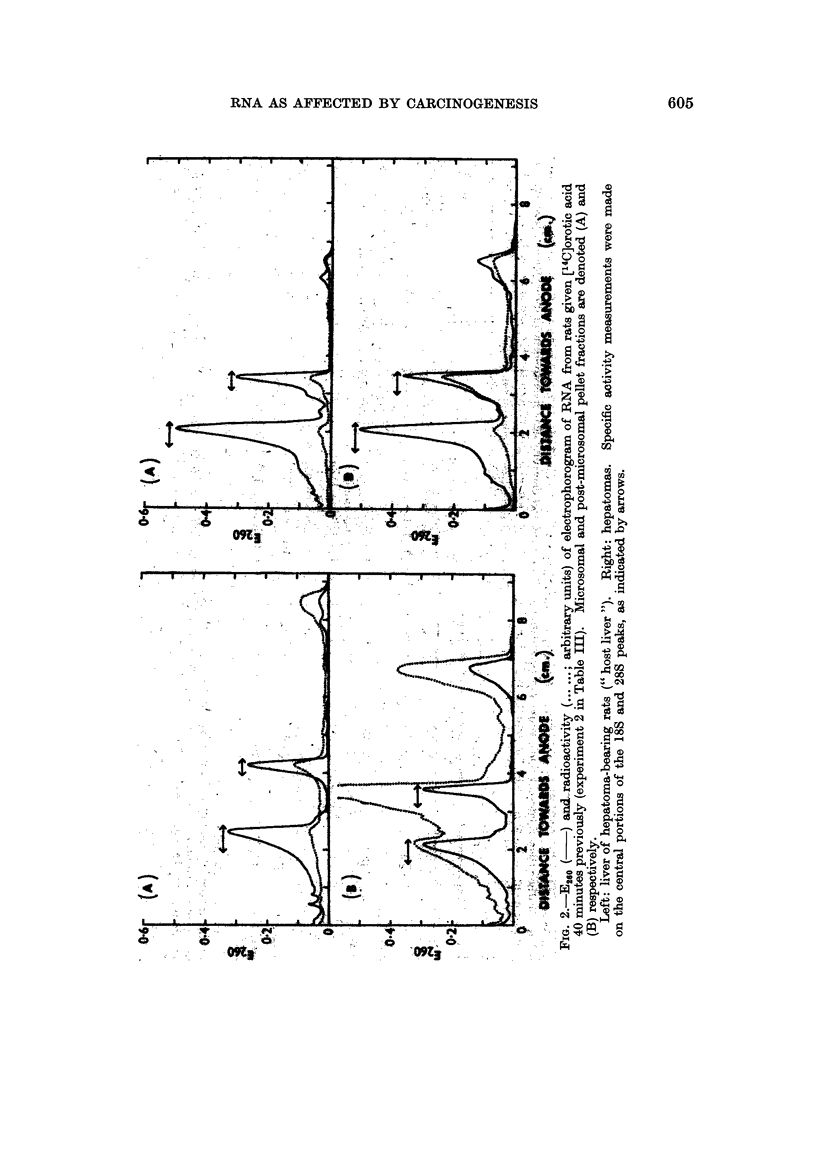

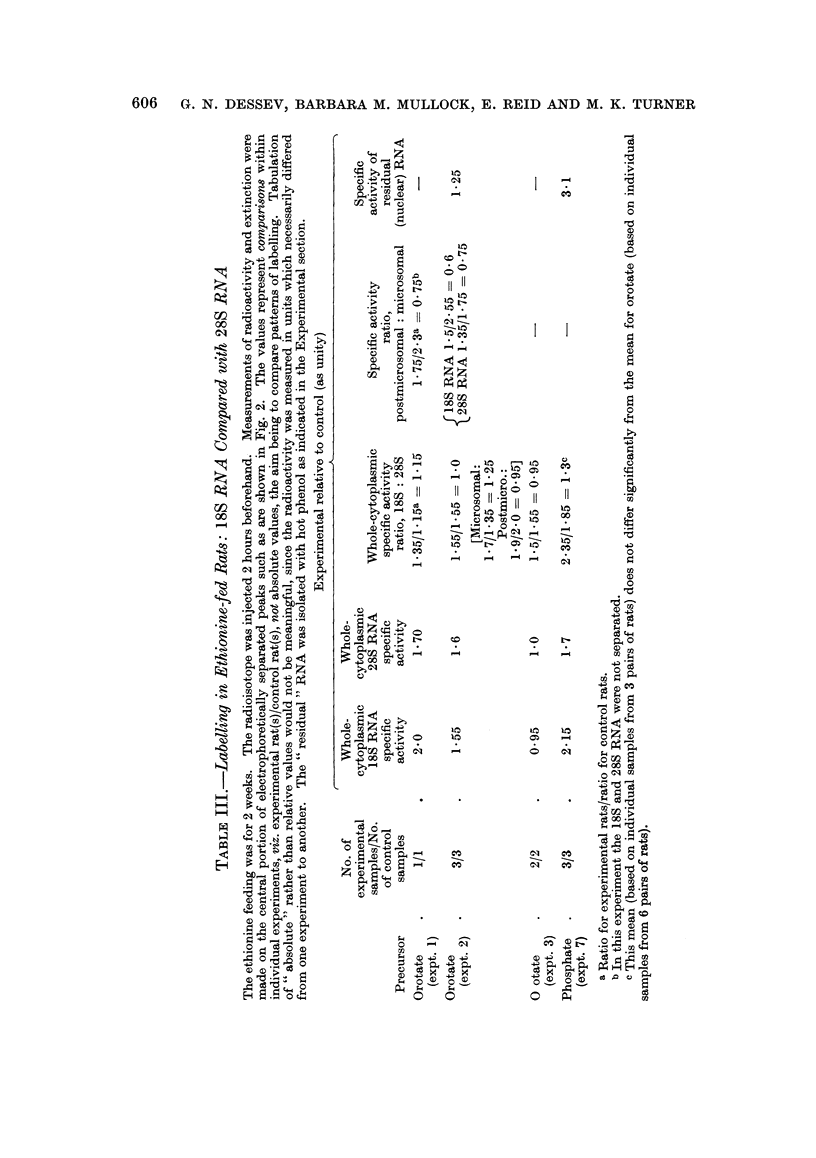

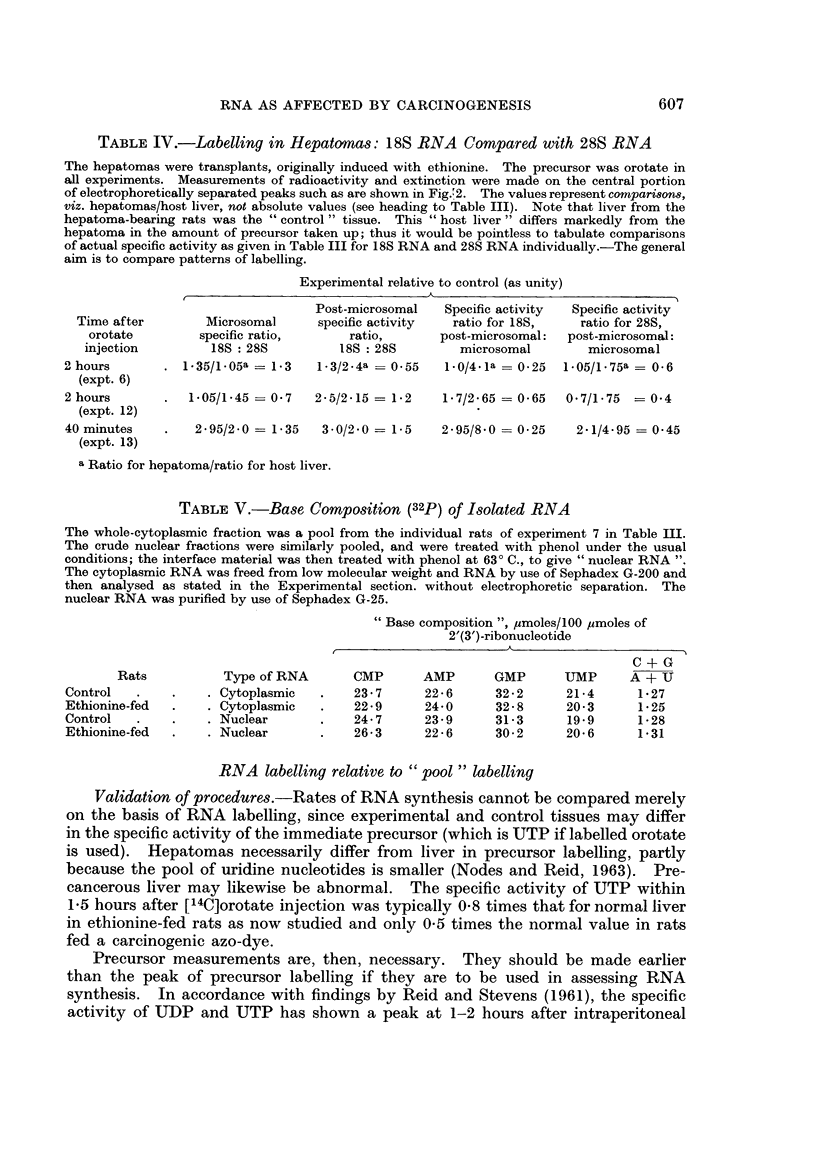

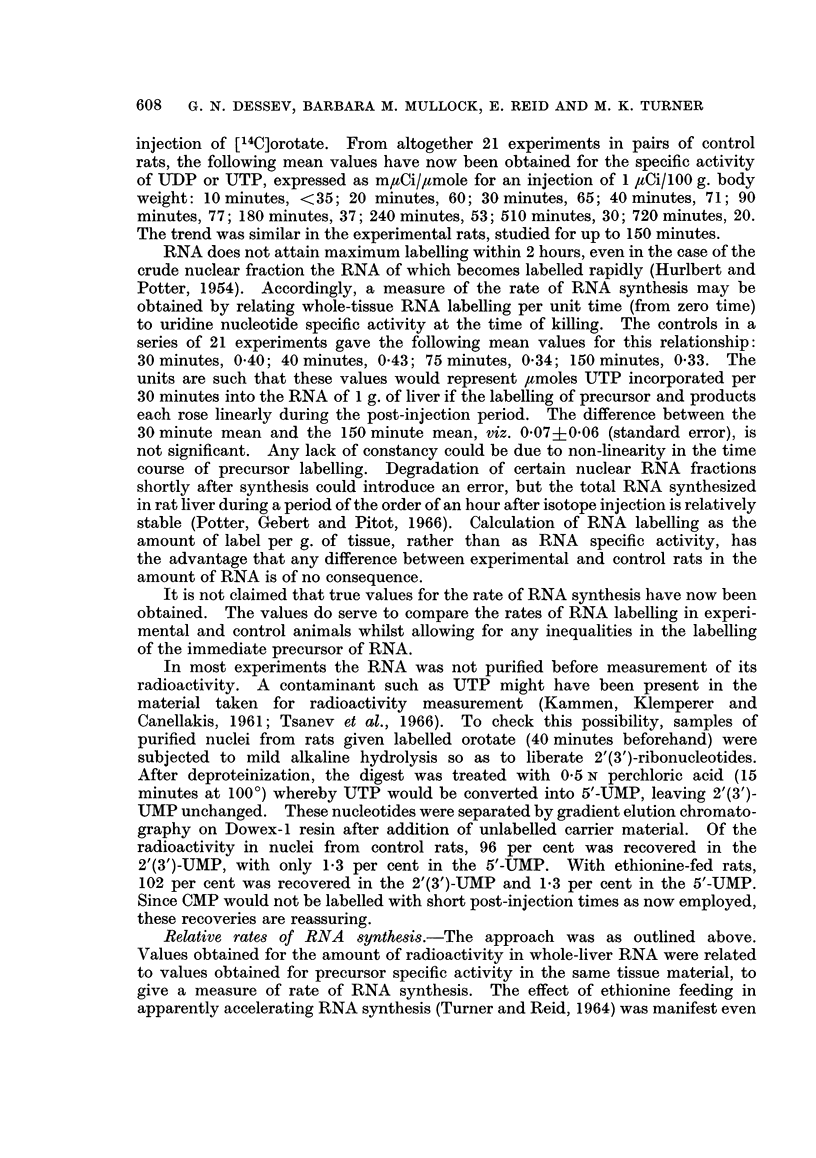

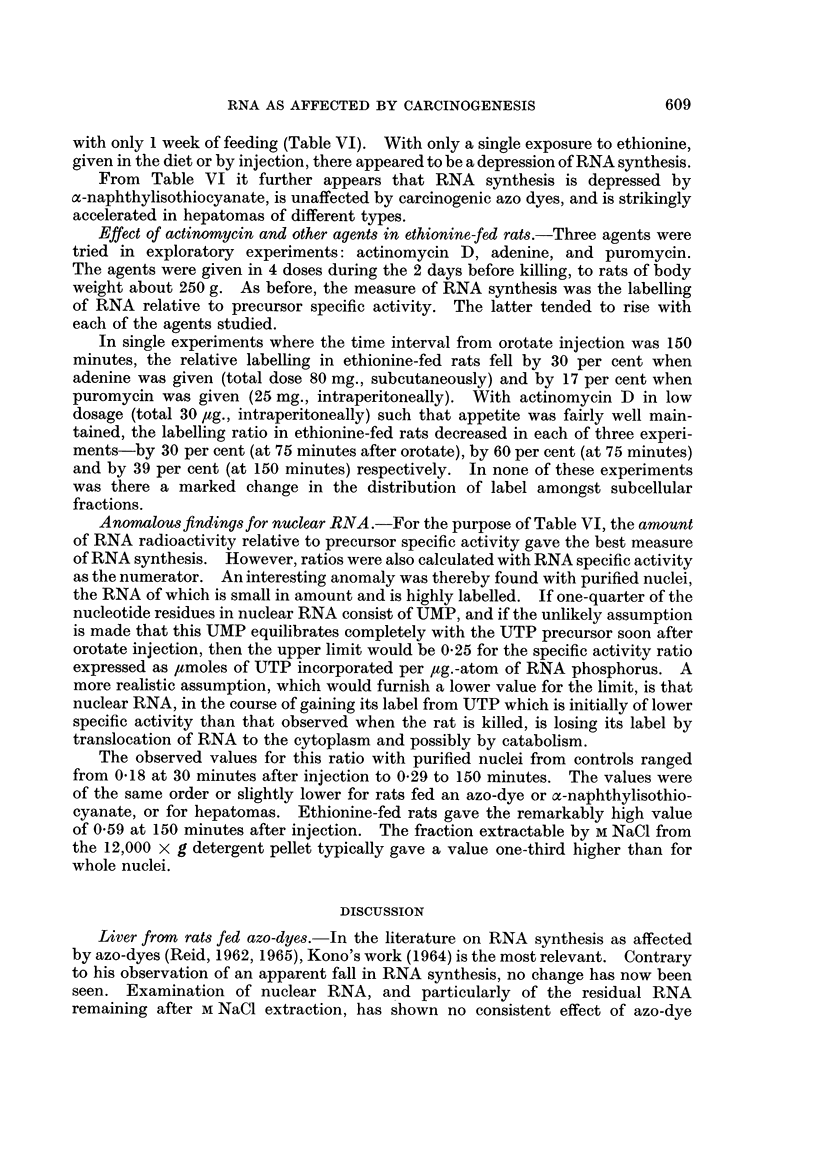

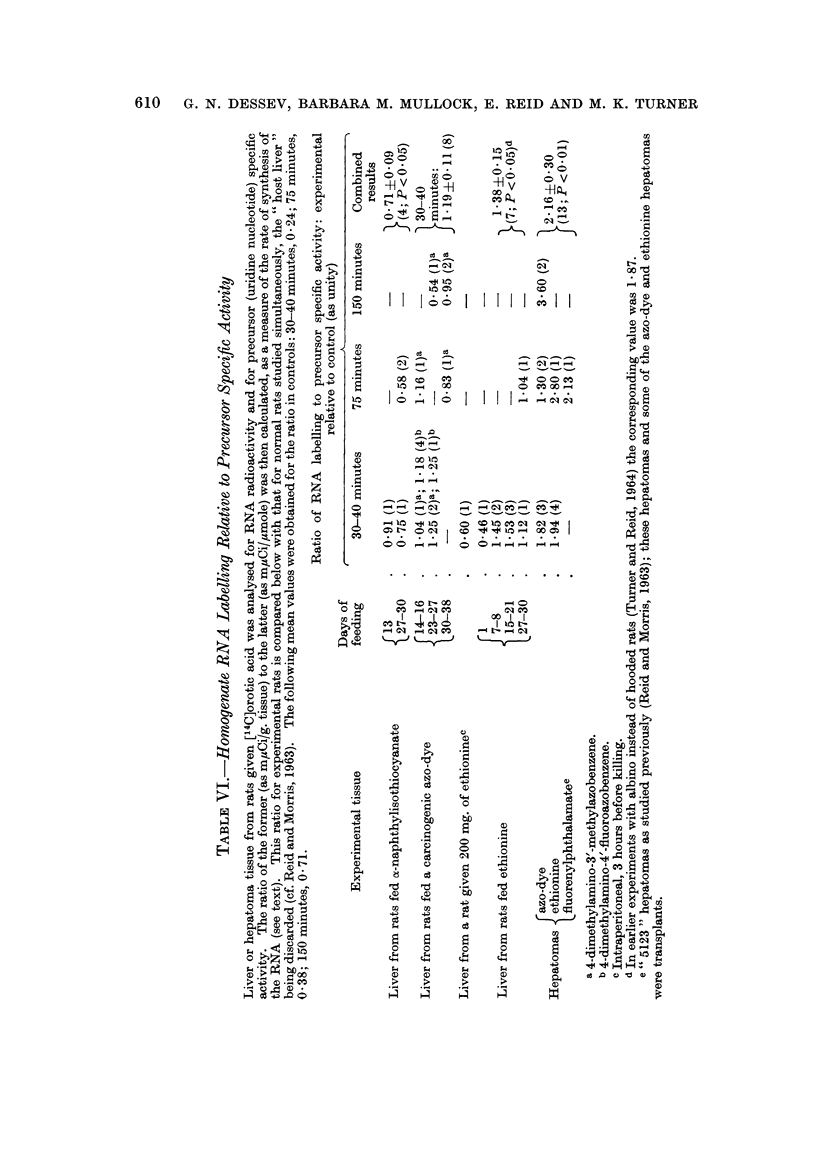

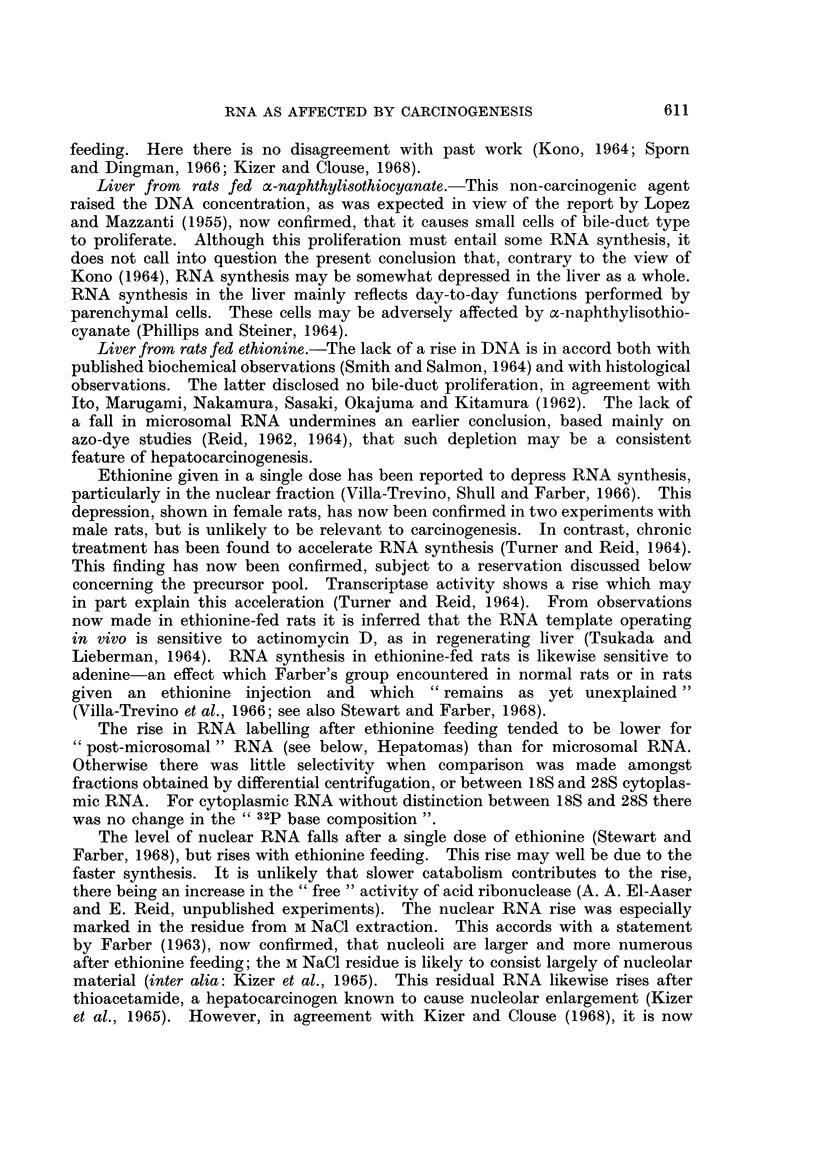

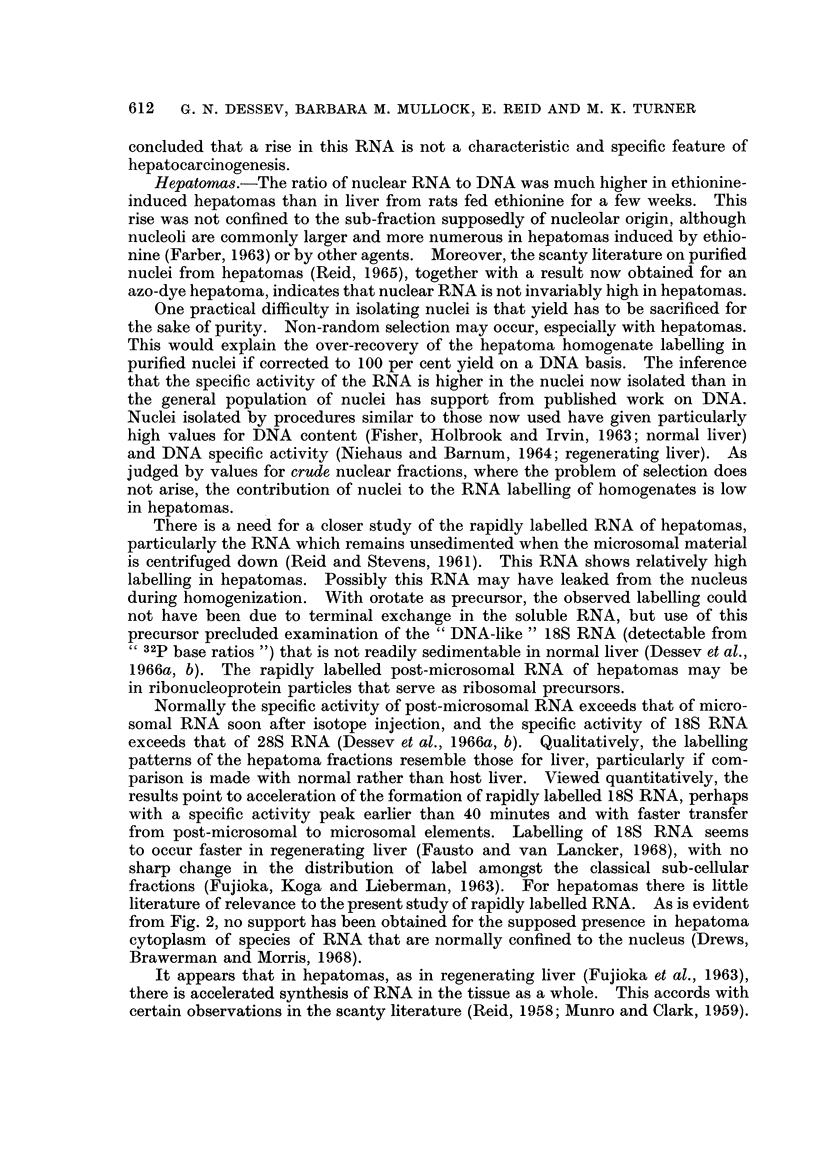

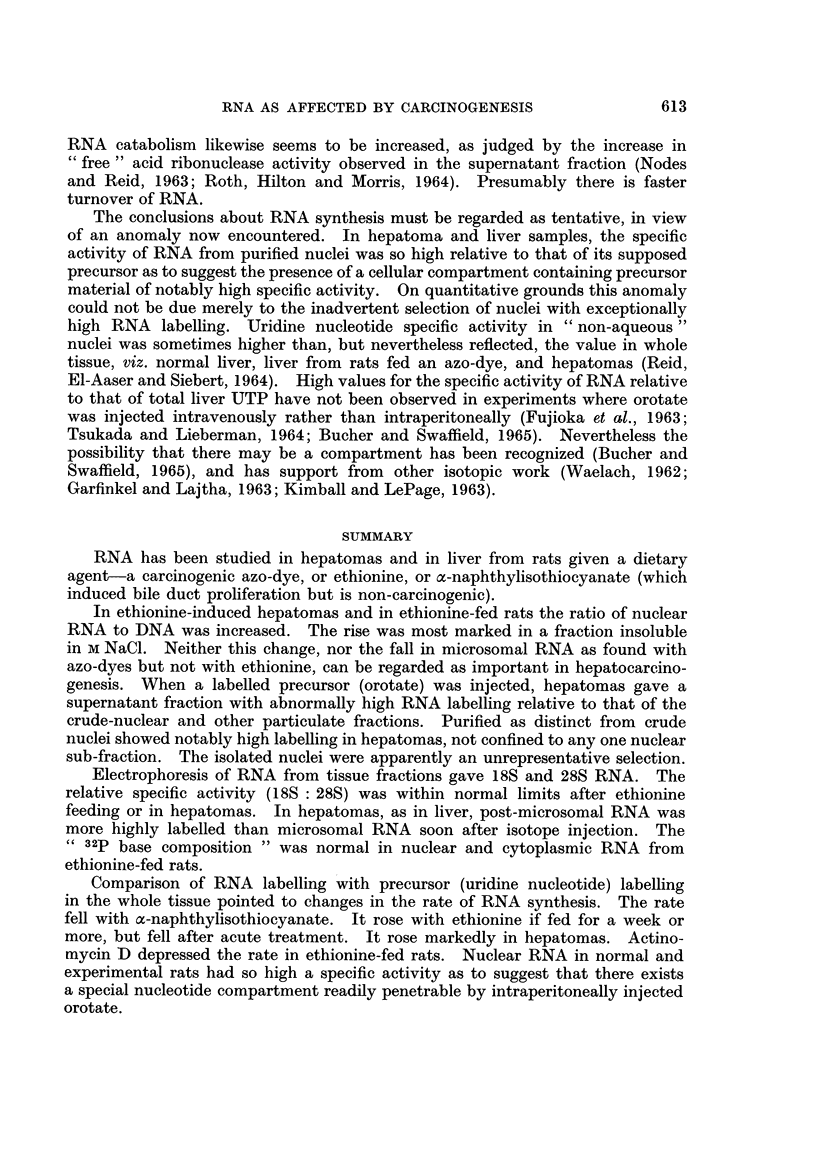

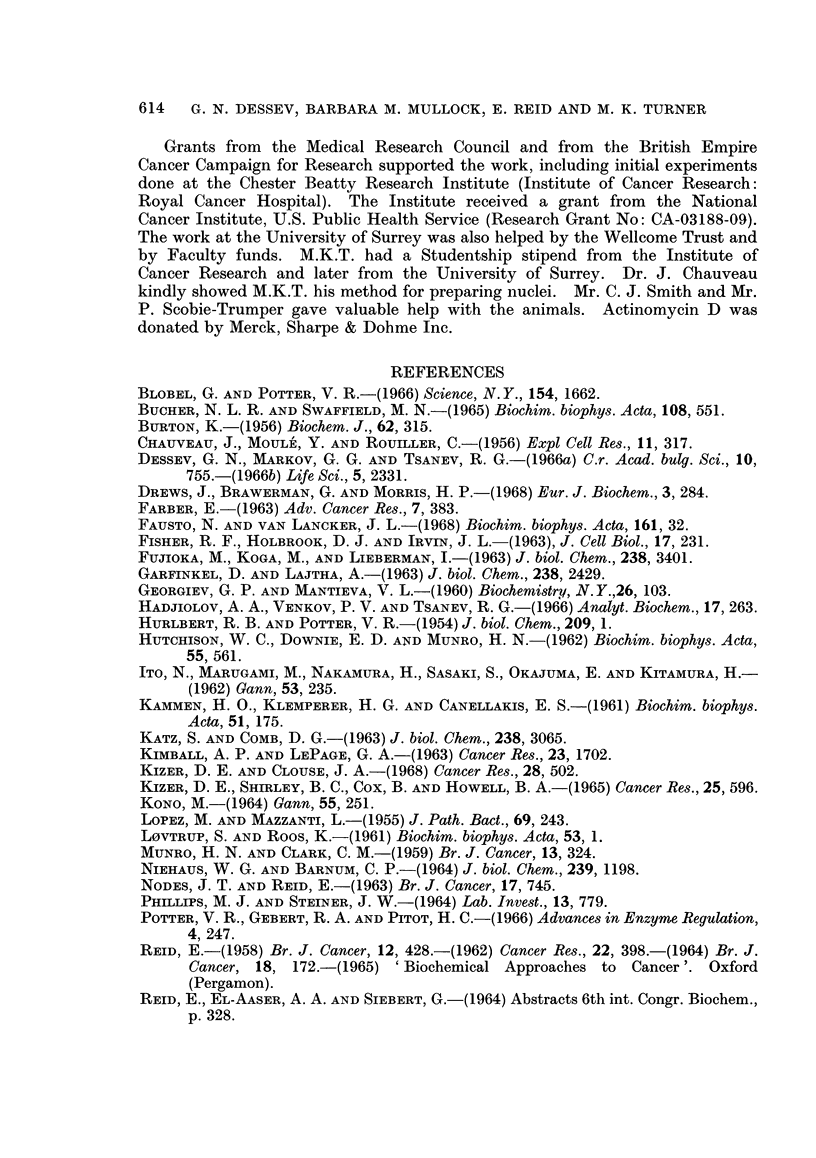

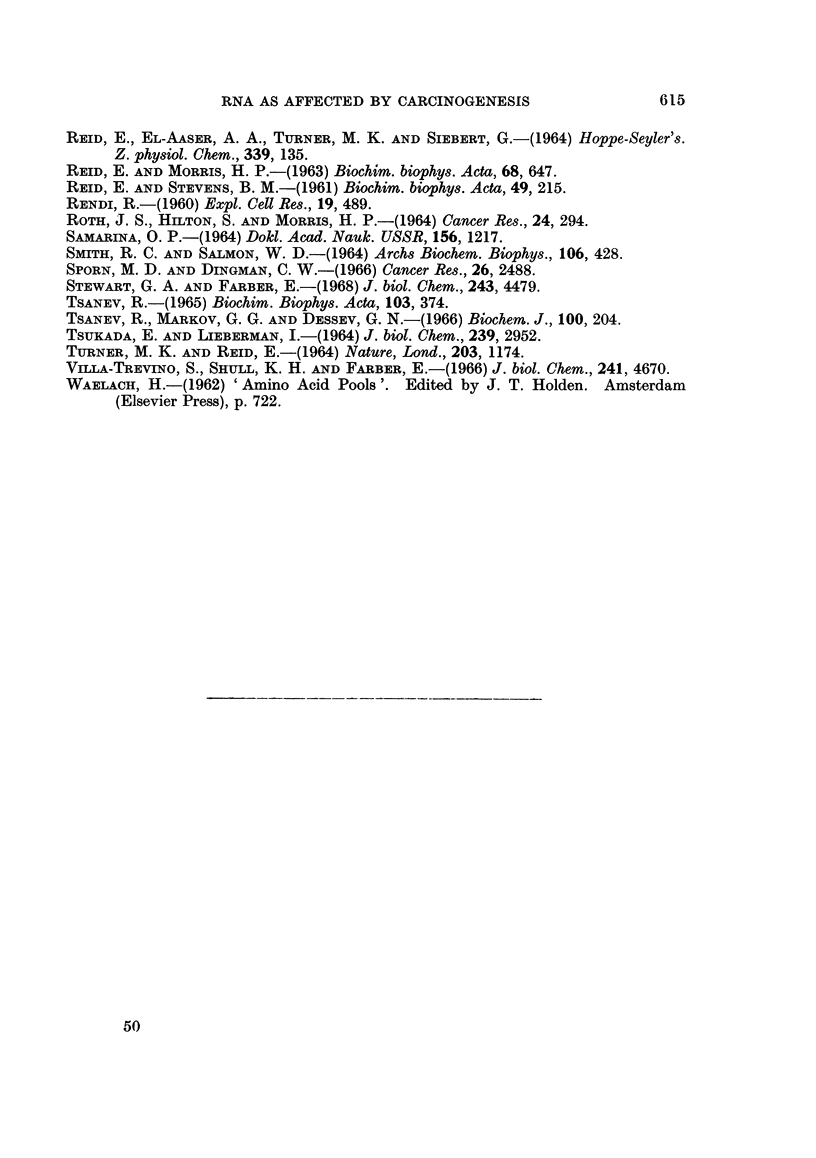

